# An approach for handling imbalanced datasets using borderline shifting

**DOI:** 10.1038/s41598-026-39118-x

**Published:** 2026-03-04

**Authors:** Mohammed G. Malhat, Alaa M. Elsobky, Arabi EI. Keshk, Hanaa A. Abdallah, Mahmoud Hussein

**Affiliations:** 1 Present Address: Department of Computer Science, Faculty of Computers and Information, Menofia University, Shebeen Elkom, Menofia, Egypt; 2 Present Address: Department of Information Technology, College of Computer and Information Sciences, Princess Nourah bint Abdulrahman University, P.O. Box 84428, 11671 Riyadh, Saudi Arabia; 3 Computer Science Department, Faculty of Information Technology, Philadelphia University, Amman, Jordan

**Keywords:** Computational biology and bioinformatics, Engineering, Mathematics and computing

## Abstract

In supervised learning tasks, class imbalance is a persistent problem that often leads to biased classification models that prioritize the majority class over the minority. To tackle this problem, we present a new resampling method called Bor- derline Shifting, which strengthens the model’s capacity to distinguish between classes close to the decision boundary by selectively enhancing significant borderline instances. Using a variety of 30 benchmark imbalanced datasets, this study com- pares the proposed method to 7 popular resampling techniques: Random Under Sampling (RUS), Random Over Sampling (ROS), SMOTE, Borderline-SMOTE, NearMiss, SMOTE-Tomek, and SMOTEENN. Performance was assessed using three well-known classifiers: Random Forest (RF), Naïve Bayes (NB), and Support Vector Machine (SVM). Evaluation metrics in- cluded F1-score, G-mean, AUC, recall, and precision. The findings show that the Borderline In every metric and classifier, the shifting method continuously produced better results. Our approach outperformed conventional methods like SMOTE and Borderline-SMOTE, achieving an average F1-score of 0.83 ± 0.06, G-mean of 0.86 ± 0.05, and AUC of 0.89 ± 0.04 with SVM. Our method significantly improved the F1-score from 0.62 (baseline) to 0.78 ± 0.07 and the AUC from 0.68 to 0.84 ± 0.06 of Naïve Bayes, which is usually sensitive to data imbalance. The robust Random Forest also benefited greatly: our approach produced the highest overall G-mean of 0.88 ± 0.04 and a stable AUC of 0.91 ± 0.03 with little variation between datasets. These findings show that the suggested Borderline Shifting approach not only solves the imbalance issue more successfully than current approaches but also improves classification performance in a consistent manner across various learning models. For real-world imbalanced learning scenarios, this makes it a viable and broadly applicable solution.

**Supplementary Information:**

The online version contains supplementary material available at 10.1038/s41598-026-39118-x.

## Introduction

With the rising use of machine learning and data mining across many industries, working with real-world data presents several challenges, one of the most common being the issue of class imbalance. This happens when the number of instances in one class (the majority) significantly outweighs those in another (the minority)^1,2^. While the majority class might represent most of the data, the minority class often carries crucial information. Unfortunately, these rare instances are sometimes misinterpreted as noise and are overlooked during model training^3^.

A good example of this is seen in customer churn prediction, especially in the telecom sector. In this scenario, it’s often more valuable for companies to retain existing customers than to attract new ones^4^. However, churn cases usually make up a very small portion of the data, sometimes only 5%. If ignored, these rare but meaningful cases can cause a model to reach seemingly high accuracy by just predicting the majority class. This may look good numerically but can lead to biased results and poor decision-making^5^.

Class imbalance poses a serious problem because most traditional classification algorithms were originally designed as- suming that all classes are roughly equal in size. As a result, when applied to skewed datasets, these models often end up favoring the majority class, making them less effective at detecting the minority class^6,7^. In extreme cases, the classifier may not recognize the minority class at all^8^.

To tackle this, researchers typically take one of two main paths. The first is to modify the learning algorithm by assigning more importance or cost to the minority class, a method known as cost-sensitive learning. The second approach involves adjusting the data itself. This includes three main strategies: oversampling, where more examples are added to the minority class; undersampling, where examples are removed from the majority class; and hybrid methods, which combine both^9–11^. While numerous resampling techniques such as Synthetic Minority Oversampling Technique (SMOTE), Borderline-SMOTE, and hybrid methods have been proposed to mitigate class imbalance, they often overlook the issue of class overlapparticularly around decision boundaries where most misclassifications occur. This paper addresses this overlooked challenge by proposing the Borderline Shifting method, which enhances the representation of critical borderline instances. This improves the model’s ability to distinguish between classes without introducing noisy or overlapping samples. The proposed method is evaluated across multiple datasets and classifiers to demonstrate its superiority over existing approaches. This paper introduces a simple yet effective method aimed at better handling class imbalance. The approach focuses on two main ideas:

First, it identifies the critical boundary area where the majority and minority classes meet, using neighborhood information.

Second, it generates new samples for the minority class, but only near this boundary, where they are most useful for improving classification.

We tested our method on a wide range of datasets30 real-world, and compared it against several other popular techniques. The results show that our method performs well, especially in generating useful synthetic data without intruding into the majority class space. When evaluated using AUC (Area Under the Curve), our approach consistently achieved better results across multiple models.

Imbalanced datasets pose significant challenges in machine learning, especially for minority class prediction. Various resampling techniques have been proposed, yet they often generate noisy samples or fail to improve classifier robustness.

The main contributions of this study are summarized as follows:

- Proposed **Borderline Shifting Oversampling (BSO)** to handle class imbalance by reclassifying borderline/noisy ma- jority instances and generating safe synthetic minority samples.
- Conducted comparative evaluation against seven existing resampling techniques on 30 benchmark imbalanced datasets.
- Demonstrated improved performance across SVM, Naive Bayes, and Random Forest classifiers using multiple metrics (AUC, F1-score, G-mean, Recall, Precision).
- Showed that BSO effectively reduces class overlap and noise, enhancing predictive accuracy and classifier robustness.

The structure of this paper is as follows: Section "Related work" gives an overview of related work. Section "The proposed approach" describes the proposed method in detail, along with the datasets and evaluation tools. Section "Results" presents the results and compares them with other methods. Finally, Section "Conclusion" offers conclusions, and Section "Future work" suggestions for future work.

## Related work

Addressing the challenges posed by noisy and ambiguous samples, particularly those near decision boundaries, has been the focus of numerous studies in the field of imbalanced learning. Researchers have developed various oversampling strategies aimed at enhancing classification performance by selectively generating synthetic data in regions that are most susceptible to misclassification.

One widely adopted technique is Borderline-SMOTE, introduced by^12^, which targets minority class samples located close to the decision boundary. These samples are considered more influential in shaping the classifier’s performance, as they are more likely to be misclassified. By oversampling such critical instances, Borderline-SMOTE helps reinforce the minority class representation where it matters most.

Another notable method is ADASYN, proposed by^13^. This technique focuses on adaptively generating synthetic data for minority instances that are harder to classify. ADASYN increases the sample density in more complex regions of the feature space, helping the model to better learn difficult patterns and refine its decision surface.

More recently, a method called Instance Density-based Hybrid Resampling (IDHR) has been proposed to further improve the quality of synthetic samples. IDHR uses the concept of local instance density to guide the generation of minority samples and incorporates a filtering mechanism to exclude those located too close to the majority class, thereby reducing the risk of generating noisy data. Comparative experiments across several datasets have shown that IDHR often outperforms standard oversampling techniques in terms of both AUC and F1-score^14^.

Other methods like Majority Weighted Minority Oversampling Technique (MWMOTE), introduced by Barua and later improved by^15^, identify key minority instances by evaluating their proximity to majority class samples. These informative instances are then used to generate synthetic examples through a clustering-based approach, improving the overall robustness of the classifier.

In addition to focused oversampling strategies, researchers have explored hybrid techniques that combine data generation with noise filtering. Methods such as SMOTE-Tomek Links, SMOTE combined with Edited Nearest Neighbors (SMOTE- ENN), Kalman-SMOTE, and SMOTE integrated with the Iterative Partitioning Filter (SMOTE-IPF) aim to clean the dataset by removing ambiguous or mislabeled instances while still enriching the minority class. These techniques apply filters like Tomek Links, Kalman smoothing, iterative partitioning, or Edited Nearest Neighbors (ENN) after oversampling. While hybrid.

approaches can offer superior performance, they often require careful parameter tuning. Poor configurations may eliminate valuable data, potentially reintroducing class imbalance or shifting the decision boundary unfavorably.

Recent research has increasingly focused on reducing the generation of noisy and overlapping samples during resampling. Douzas and Bacao (2019)^16^ proposed *Geometric SMOTE*, which enhances the interpolation mechanism with geometric con- straints to generate more realistic synthetic instances. Liu et al. (2020)^17^ introduced a constrained oversampling strategy that restricts sample generation to safe regions to minimize noise. Salam and Cengiz (2022)^18^ integrated noise detection with a boosting procedure to develop a more robust oversampling approach, while Li and Zhu (2023)^19^ presented *OALDPC*, an oversampling method based on local density peaks clustering that preserves the original data distribution.

Chen, Guo, and Mao (2024)^20^ further proposed an adaptive oversampling method that simultaneously performs cluster- ing and noise filtering to alleviate overlap-related misclassification. More recently, Li and Liu (2025)^21^ introduced a hybrid sampling algorithm combining natural neighbors with density estimation to effectively handle both class imbalance and over- lapping regions. In addition, Liu et al. (2023)^22^ developed a noise-robust oversampling framework based on unsupervised learning to improve classification performance under high noise conditions, and Sun et al. (2025)^23^ combined geometric modeling with noise detection to enhance minority class representation.

Collectively, these studies highlight a clear trend toward integrating geometric constraints, density-based analysis, and noise control within oversampling frameworks. However, most existing approaches primarily focus on local geometry or density estimation. In contrast, the proposed *Borderline Shifting* method adaptively refines the decision boundary between overlapping classes by reclassifying ambiguous samples away from overlap regions, thereby improving classifier generaliza- tion.

The following subsections detail several commonly used resampling techniques. Section "Resampling technique" introduces resampling strate- gies that divide to Oversampling technique such as Random Oversampling, SMOTE, and its variants , under-sampling meth- ods, including Random Undersampling and NearMiss, and hybrid techniques that blend both under- and oversampling, like SMOTE-Tomek and SMOTENN. Finally, Section "Imbalanced Learning" provides an overview of learning algorithms specifically designed to handle imbalanced data.

### Resampling technique

Several resampling methods have been developed to handle class imbalance by adjusting the distribution of minority and majority samples. The following techniques are considered in this study.

#### Oversampling technique

This section outlines three commonly used oversampling methods: Random Oversampling, SMOTE, and Borderline- SMOTE.

**Fig. 1:**
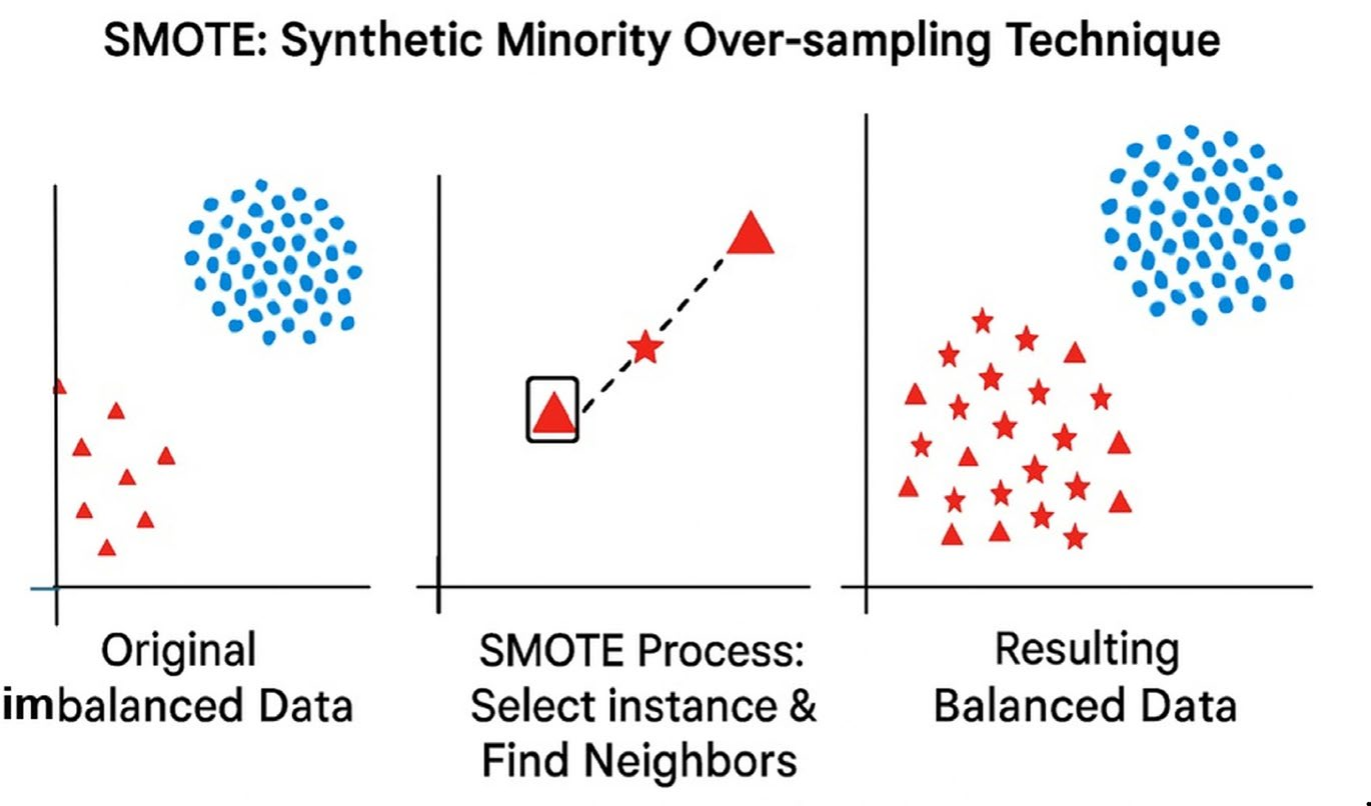
Illustration of the Synthetic Minority Over-sampling Technique (SMOTE). The left panel shows the original imbalanced dataset, where minority class samples (red triangles) are underrepresented relative to the majority class (blue circles). The middle panel depicts the SMOTE process, in which a minority instance is selected and synthetic samples are generated along the line segments joining its nearest minority neighbors. The right panel presents the resulting balanced dataset after oversampling, demonstrating how SMOTE increases minority class density to reduce class imbalance^27^ and quick technique, it may lead to the loss of important information. As discussed by^28^, its effectiveness depends on maintaining representative samples from the majority class during reduction.

1. Random Oversampling: This is a simple method where instances from the minority class are duplicated randomly to balance the class distribution. As described by^24^, the technique increases the representation of the minority class by repeatedly adding existing samples. Although effective in balancing the dataset, it can lead to overfitting, as it does not introduce any new information, only repeated data points.
2. SMOTE (Synthetic Minority Oversampling Technique): SMOTE improves upon random oversampling by generating new, synthetic samples instead of merely duplicating existing ones. According to^25,26^, the algorithm selects a minor- ity class instance and generates new samples along the line segments joining that instance and its k nearest minority neighbors. This interpolation creates more diverse training data, helping the model learn better boundaries between the classes. Fig. 1. illustrates this concept by showing how synthetic instances are created between neighboring minority samples.
3. Borderline-SMOTE: Borderline-SMOTE refines the SMOTE approach by focusing specifically on samples near the decision boundary, where misclassification is more likely. As discussed by^12^, the algorithm identifies borderline in- stancesthose minority samples surrounded by a higher number of majority class neighborsand generates new samples in these critical regions. It avoids creating synthetic data for “safe” or “noisy” points, instead targeting instances that fall within a sensitive margin between the classes. A sample is considered borderline if the number of majority neighbors lies between half and the total number of its M nearest neighbors. Once the borderline minority instances are isolated, new samples are generated by interpolating between them and their minority neighbors.

#### Under sampling techniques

Under-sampling techniques aim to reduce the number of majority class instances to balance the dataset. This section highlights two commonly used approaches: Random Under-Sampling and NearMiss. 1. Random Under-Sampling: This method reduces the size of the majority class by randomly removing samples. The process is repeated until the number of majority instances is balanced with the minority class. While it is a simple.

1. Random Under-Sampling: This method reduces the size of the majority class by randomly removing samples. The process is repeated until the number of majority instances is balanced with the minority class. While it is a simple.

2. NearMiss: NearMiss is a more strategic approach that considers the distance between instances. It selects the majority class examples that are closest to the minority class instances. By focusing on those near the decision boundary, it helps refine the classification margin. According to^29^, NearMiss chooses samples with the smallestdistances to the minority class, enhancing class separability without random deletions.

#### Combination of over-sampling and under-sampling techniques

Hybrid methods combine the strengths of both over-sampling and under-sampling strategies to handle class imbalance more effectively. Two well-known hybrid techniques are SMOTE-Tomek and SMOTE-ENN.

**Fig. 2:**
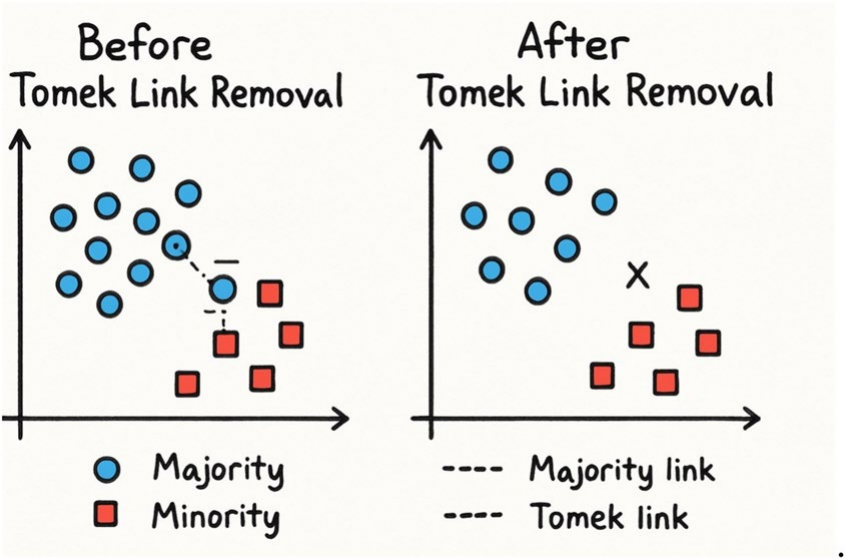
Illustration of the Borderline-Tomek process used to reduce class overlap. The figure shows how Tomek links are applied to identify pairs of nearest samples from opposite classesone minority and one majority instancebased on Euclidean distance. When a Tomek link is detected, the majority instance closest to the minority sample is removed, thereby cleaning the decision boundary and eliminating borderline noise. This step enhances class separability and prepares the data for subsequent oversampling^33^.

**Fig. 3:**
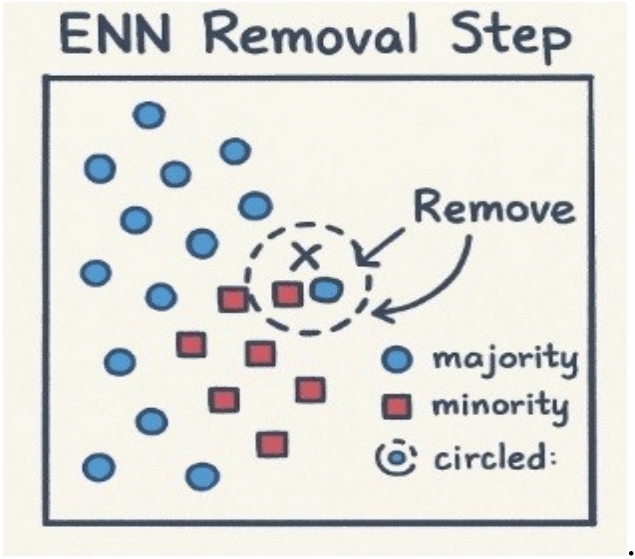
Illustration of the SMOTE-ENN process for identifying and removing noisy or overlapping samples based on their nearest neighbors. After applying SMOTE to generate synthetic minority samples, the Edited Nearest Neighbor (ENN) rule examines each instance and its surrounding neighbors using Euclidean distance. Samples whose class labels differ from the majority of their neighbors are considered noisy or misclassified and are subsequently removed. This combined oversamplingcleaning approach enhances boundary definition and reduces class overlap before model training^27^.

1. SMOTE-Tomek: This method begins by applying SMOTE to generate synthetic samples for the minority class. Then, it uses Tomek links to clean the overlapping regions by identifying and removing ambiguous examples that are close to the decision boundary. As described by^30^, this combination helps improve class separability and reduces noise, leading to better model performance. Fig. 2. illustrates how Tomek links eliminate samples from regions where the class distinction is unclear^30^.
2. SMOTE-ENN: It combines over-sampling through SMOTE and under-sampling using the Edited Nearest Neighbor (ENN) algorithm. First, SMOTE generates synthetic data points by interpolating between a minority instance and its k nearest neighbors. Then, ENN removes noisy or misclassified instances from the dataset based on KNN distance calculations. If a samples class label differs from the majority of its nearest neighbors, it is removed. This dual approach improves both the representation and quality of the training data^31^ explain that this method not only balances the dataset but also refines it by eliminating borderline or mislabeled data points. Fig. 3. demonstrates how the technique leverages KNN to assign new samples to the appropriate class and filter out inconsistencies.

#### Comparative evaluation of existing resampling methods

Several resampling strategies have been proposed to address class imbalance^32^. Each technique offers unique benefits but also introduces certain limitations that may hinder its generalizability or effectiveness in specific scenarios. Table1. provides a critical evaluation of the primary methods considered in our study:

**Table 1:** Strengths and weaknesses of existing resampling techniques.

Technique	Strengths	Weaknesses
Random Undersampling (RUS)	Simple and fast; effectively reduces training time	Discards potentially useful majority class instances, leading to information loss and reduced generalization
NearMiss	Retains informative majority instances close to minority samples	Highly sensitive to noise; may under- represent the majority class boundary
Random Oversampling (ROS)	Easy to implement; no data is removed	Prone to overfitting due to repeated du- plication of minority samples
SMOTE	Creates synthetic samples to improve class balance and recall	May introduce noise or overlapping samples, distorting class boundaries
Borderline-SMOTE	Focuses on generating synthetic sam- ples near the decision boundary	Cannot control majority overlap; may introduce ambiguity near class borders
SMOTE-Tomek(Hy- brid)	Reduces class overlap and noise through Tomek link removal	Tomek removal may discard informa- tive borderline instances
SMOTEENN (Hybrid)	Improves noise removal and boundary clarity using Edited Nearest Neighbor	May be too aggressive; risks removing valuable samples and increasing com- plexity

This evaluation underscores that while each method has merits, none fully address both borderline sensitivity and class overlap simultaneously. This limitation served as the primary motivation for developing our proposed Borderline Shifting ap-proach, which aims to reinforce decision boundary learning by emphasizing informative borderline samples while minimizing majority class interference.

### Imbalanced learning

Imbalanced learning^34^ deals with classification problems where one class significantly outweighs the others in terms of sample size. This imbalance often leads to biased predictions, where models are overly inclined toward the majority class. To counter this issue, researchers have applied a variety of machine learning algorithms across multiple real-world domains.

For example^35^, introduced a predictive framework for flight delays that integrates both temporal and spatial factors. By leveraging a Random Forest (RF) classifier, their model effectively captured the interplay between weather conditions, traffic patterns, and the aviation networks structure, achieving high predictive accuracy.

In the field of sentiment analysis^36^, analyzed 4,000 Facebook reviews of Karachi restaurants using various classifiers, including Naïve Bayes (NB), Logistic Regression, Support Vector Machines (SVM), and Random Forest. Among these, the Random Forest model delivered the highest accuracy, reaching 95%.

Similarly^37^, examined sentiment classification using 1,000 Bengali-language restaurant reviews. They tested Decision Trees, Random Forest, and Multinomial Naïve Bayes, concluding that the Multinomial NB algorithm was most effective, achieving 80.48% accuracydemonstrating its robustness even in imbalanced textual data.

Beyond text classification, imbalanced learning has proven crucial in accident detection and response systems.

Research by^38–41^ underscores the value of timely and accurate detection of rare events. For instance, Parsa et al. worked with a dataset containing over 85,000 entries, of which only 32 were actual accidents. To address the imbalance, they employed SMOTE (Synthetic Minority Oversampling Technique) to synthetically generate minority instances, which significantly im- proved the models ability to detect these rare yet critical events.

Moreover, classifiers like Probabilistic Neural Networks (PNN) and SVMs have shown impressive results in such scenarios. Notably, Parsa et al. (2019) demonstrated that PNN could achieve up to 90% prediction accuracy for accident detection as early as five minutes before occurrence, highlighting the potential of advanced models in real-time applications.

While these studies showcase the effectiveness of combining machine learning models with resampling strategies, they also reveal a persistent limitation: many current approaches do not adequately address class boundary regions. Traditional oversampling techniques such as SMOTE, Tomek Links, and Edited Nearest Neighbors (ENN) often overlook the geometric complexities near decision boundaries, where the most critical classification errors occur.

Although SMOTE and its extensions have proven effective in reducing class imbalance, they do not adequately address the issue of class overlap, which often leads to poor generalization in real-world data. Borderline-SMOTE attempts to focus on samples near the decision boundary but lacks mechanisms to suppress noise or reduce overlap from the majority class. Hybrid methods such as SMOTE-Tomek and SMOTEENN improve this by incorporating cleaning strategies, but they may aggressively remove informative samples. In contrast, our proposed Borderline Shifting method is designed to carefully en- hance informative borderline instances while maintaining decision boundary clarity, directly addressing both class imbalance and class overlap.

Several studies have attempted to address class imbalance in tandem with class overlap, recognizing that overlap near the decision boundary exacerbates misclassification risk. Hybrid techniques such as SMOTE-ENN and SMOTE-Tomek aim to both balance the dataset and reduce overlapping regions by combining oversampling with noise cleaning methods. While effective, these can sometimes remove valuable borderline instances. More advanced methods like ADASYN prioritize gen- erating synthetic samples in complex regions, but may amplify noise if not properly constrained. Margin-based methods such as Majority Distribution Oversampling (MDO) focus on maximizing class separation but are often computationally intensive. These challenges highlight the need for methods that can emphasize informative borderline cases while minimizing overlap a gap our proposed Borderline Shifting method seeks to address.

To bridge this gap, this paper introduces a novel approach that repositions decision boundaries by reclassifying majority class instances near minority class samples. Utilizing a K-Nearest Neighbors (KNN)-based technique, our method identifies and adjusts borderline majority instances and strategically generates synthetic minority samples. This boundary-aware strategy reduces the risk of misclassifying minority cases and more accurately reflects the underlying data distribution. Ultimately, this contributes to improved model performance and a better understanding of spatial relationships in imbalanced datasets. While various resampling techniques have been proposed to address class imbalance, few specifically tackle the challenge of borderline instances.

#### Summary and research motivation

Existing resampling techniquessuch as RUS, ROS, SMOTE, and their hybridshave been widely applied to address class im- balance. However, they often fall short when handling borderline and overlapping class distributions, which are critical in determining classification performance. While SMOTE-based methods introduce synthetic diversity, they may generate noisy or ambiguous samples, particularly near decision boundaries. Moreover, very few approaches explicitly target borderline instances in a way that reduces overlap without losing minority class representation.

Unlike existing resampling techniques that either (i) detect and remove noisy samples or (ii) generate new samples near decision boundaries, the Borderline Shifting Oversampling method develops a framework that integrates these two aspects. First, it identifies the borderline minority samples and adaptively shifts them toward the nearest safe region, which can better separate the classes. Then, the method synthesizes instances around those shifted points to prevent the generation of any overlapping or noisy samples.This dual-phase mechanism distinguishes from methods such as Borderline-SMOTE, Safe- Level-SMOTE, and Geometric-SMOTE, which do not explicitly adjust the minority data distribution before synthesis.

This review reveals a research gap: most existing methods either lack focus on borderline cases or introduce excessive synthetic noise. Our proposed method, Borderline Shifting, is designed to fill this gap by focusing resampling efforts on informative borderline regions, aiming to improve both decision boundary clarity and classification robustness. The following section outlines our approach in detail.

## The proposed approach

### Data preprocessing

Before applying resampling techniques and training the classifiers, all datasets underwent a standardized preprocessing pipeline to ensure fair and meaningful evaluation^42^:

- **Data Cleaning:** Datasets were inspected for missing values, inconsistent entries, and formatting issues. Missing nu- merical values were imputed using mean substitution, and categorical inconsistencies were corrected based on domain knowledge where available^43^.
- **Normalization:** All numerical features were normalized using min–max scaling to the range [0, 1]. This step was particularly important for distance-based methods like SMOTE and SVM with RBF kernel, which are sensitive to feature scale.
- **Encoding:** For datasets containing categorical features, one-hot encoding was applied to ensure compatibility with classifiers and resampling algorithms.
- **Feature Selection:** To ensure consistent comparison across datasets, no dataset-specific feature selection techniques were applied. All original features were retained unless the dataset provider recommended otherwise.

### Data resampling

The proposed method^44^ addresses the class imbalance problem through a two-fold strategy: shifting the decision boundary and increasing minority class instances. This approach focuses specifically on refining the region near the class boundary by identifying and modifying majority instances that cause misclassification.

**Fig. 4:**
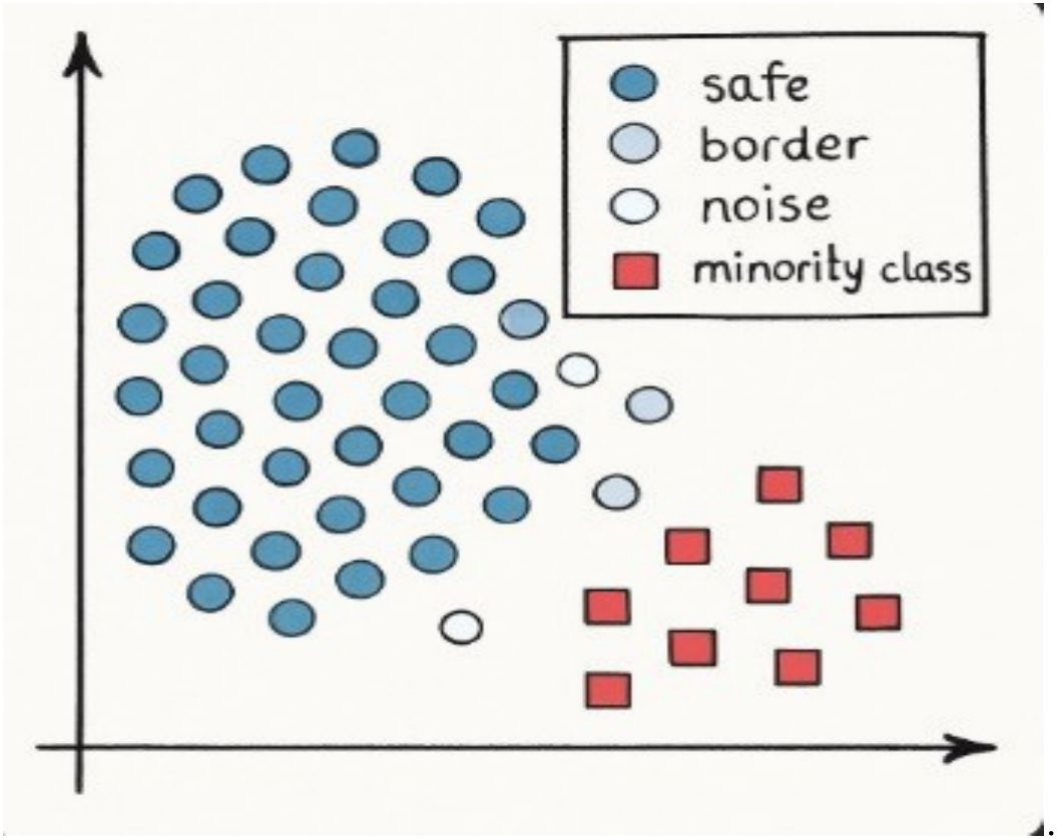
Illustration of the three majority-class regions identified during resampling. Each majority-class instance is categorized as safe, borderline, or noise based on the distribution of its neighboring minority and majority samples. Safe instances (dark blue) are surrounded by other majority samples, borderline instances (light blue) lie near the decision boundary, and noisy instances (white) are surrounded primarily by minority samples. Minority-class instances are shown in red. This classification is commonly used to guide noise handling and oversampling strategies in imbalanced learning^46^ proposed a neighbor-displacement-based oversampling approach to improve class representation in multiclass imbalance sce- narios.E, as well asSaglam (2025)^47^ introduced a noise module which relocates points for noise-tolerant synthetic point gen- eration including DatRel. In contrast to these works, our method is aimed at relocate (or reassign) majority class instances near the decision boundary to the minority class, thereby reshaping the boundary and improving classification accuracy, which provides more stable sample generation and reduces overlapping instances. Fig. 5. visually depicts the data distribution before and after the boundary shift process.

According to this method, majority class instances are categorized into three types: SAFE, BORDER, and NOISE, as illustrated in Fig. 4:

- NOISE instances are outliers that typically appear in areas dominated by minority class instances. These are uncommon and may often be the result of anomalies or mislabeling.
- BORDER instances are located near the decision boundary and are likely to overlap or interact with minority class instances, posing a high risk of misclassification.
- SAFE instances are well-separated from the minority class and represent typical, easily classified majority samples.

**Fig. 5:**
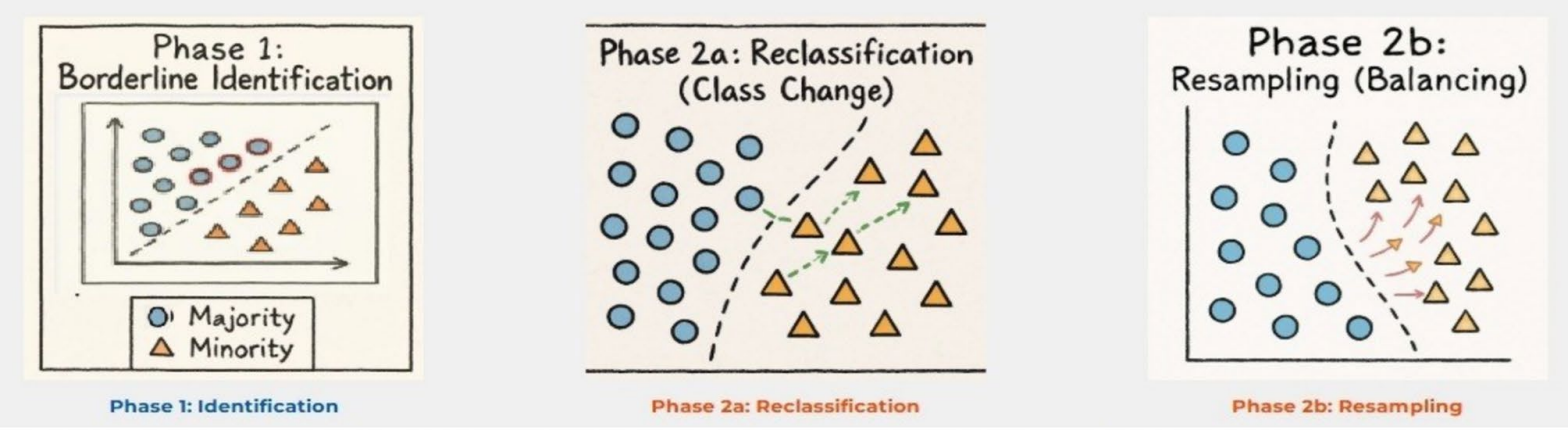
Workflow of the proposed BSO algorithm. Phase 1 identifies borderline majority-class instances near the decision boundary (red outline). Phase 2a reclassifies selected borderline instances as minority class (green arrows indicate class change). Phase 2b performs targeted resampling to generate synthetic minority instances (pink arrows) and balance the dataset. The diagram illustrates both the identification and enhancement of borderline instances to improve classifier performance.

This paper primarily focuses on BORDER and NOISE instances within the majority class. The main concept of our approachis to move samples in the feature space, such that they are better distributed across classes. This idea is consistent with the previouswork which examines data transfer technique to address class imbalance. Putrama and Martinek (2025)^45^.

**Fig. 6:**
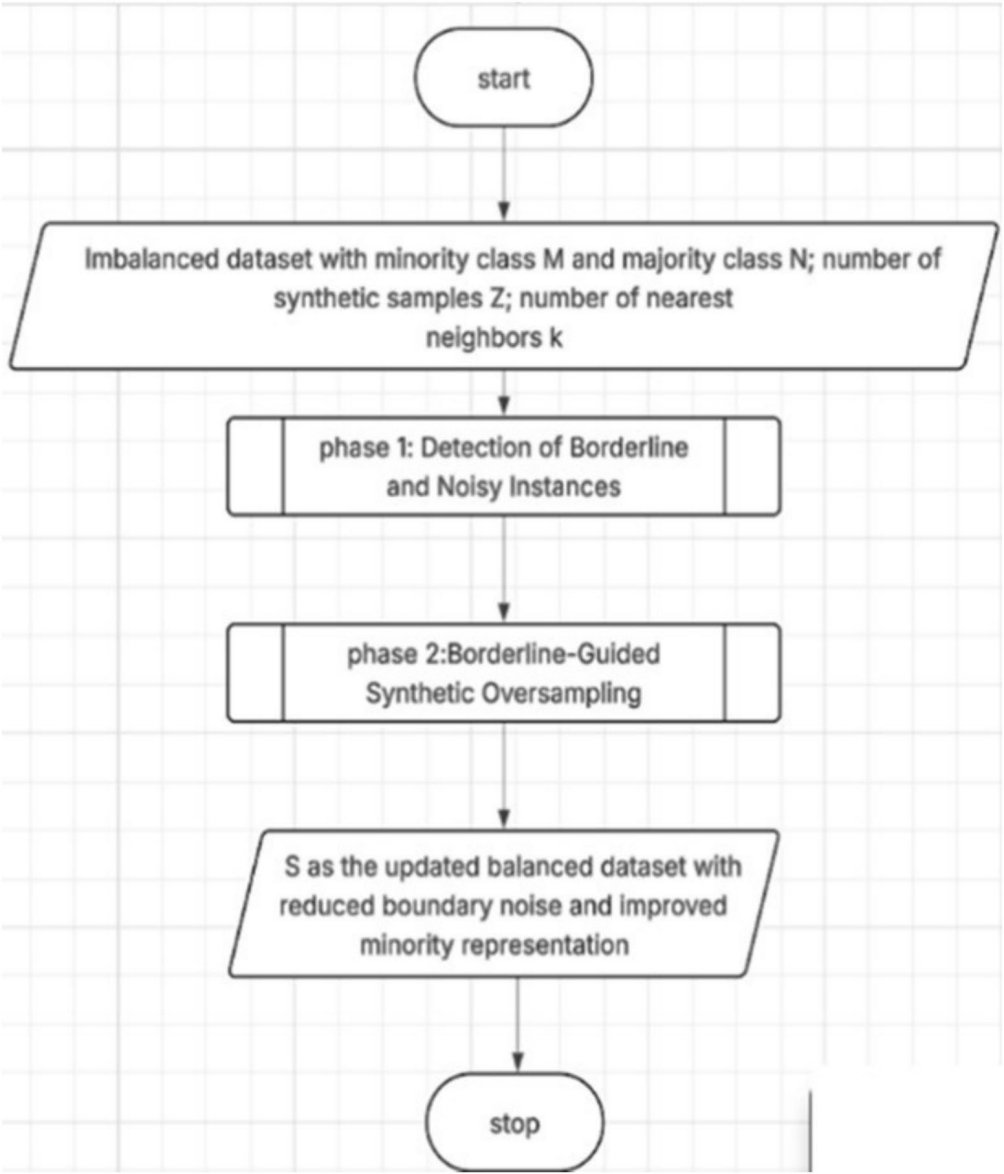
Overall workflow of the proposed Borderline Shifting and Oversampling (BSO) method, illustrating the two main phases from the initial imbalanced dataset to the final balanced output.

This study is detailed in Algorithm 1 and is represented in the flowchart (Fig. 6.),The method begins by taking as input an imbalanced dataset composed of a minority class , a majority class *N*, and a specified number of synthetic samples *Z*to be generated. The goal is to produce a balanced dataset *S* that improves classification performance through a two-step process which introduces a two-phase resampling strategy designed to address class imbalance and improve the classifiers ability to distinguish between overlapping classes. The method combines the identification of borderline and noisy instances with a targeted oversampling technique that emphasizes refinement of the decision boundary.

- Detection of Borderline and Noisy Instances
- Borderline-Guided Synthetic Oversampling

### Phase one: detection of borderline and noisy instances

In the first phase, the algorithm evaluates each instance belonging to the majority class by examining its local neighborhood using the k-nearest neighbors (k-NN) approach. For each majority class instance, the number of surrounding minority and majority instances is determined.

If the number of minority neighbors exceeds that of the majority, the instance is considered to lie near the class boundary and is classified as a borderline sample. In contrast, if no majority instances are present in the neighborhoodindicating isolation in a region dominated by the minority classthe instance is regarded as noise. These cases are flagged and added to respective lists to guide subsequent processing. This phase effectively identifies the majority class instances that either contribute noise or lie in ambiguous regions prone to misclassification (see lines 37 in Algorithm 1).

### Phase two: borderline-guided synthetic oversampling

The second phase focuses on reinforcing the representation of the minority class by generating synthetic samples in critical areas. This is achieved by applying a modified version of the Synthetic Minority Oversampling Technique (SMOTE), tailored to operate specifically on borderline minority instances identified in the previous phase.

For each minority class instance, its k-nearest neighbors from the same class are located. For example, if x is a minority instance with neighbors *x*_1_, *x*_2_, …, *x*_*i*_, a new synthetic point *x*^′^ is generated using:

1 $$x^{\prime\prime} = x_{i} + \lambda (x^{\prime}_{i} - x_{i} )$$

As λ is a random number between 0 and 1 (line 16), and *x*^′^ , and *x*_*i*_are different instances of the minority class. A synthetic data point is then generated by interpolating between the instance and one of its neighbors, such as *x*_2_, and *x*_3_, *x*_4_, and *x*_5_, etc., producing *x*_16_, *x*_25_, and *x*_4_synthetic instances, like and so forth (see Fig. 6.). These newly created points are added to the minority class (line 17). The interpolation is controlled by a randomly selected scalar between 0 and 1, which ensures variability in the synthetic samples. This process continues iteratively until the number of newly generated instances reaches a predefined target that restores balance between the classes.

By focusing on oversampling efforts on borderline regionswhere misclassifications are more likelythe method promotes better generalization and enhances the classifiers sensitivity to the minority class. Additionally, excluding noisy or isolated instances from the synthetic generation process prevents the introduction of misleading patterns into the training data.

### Outcome

**Fig. 7:**
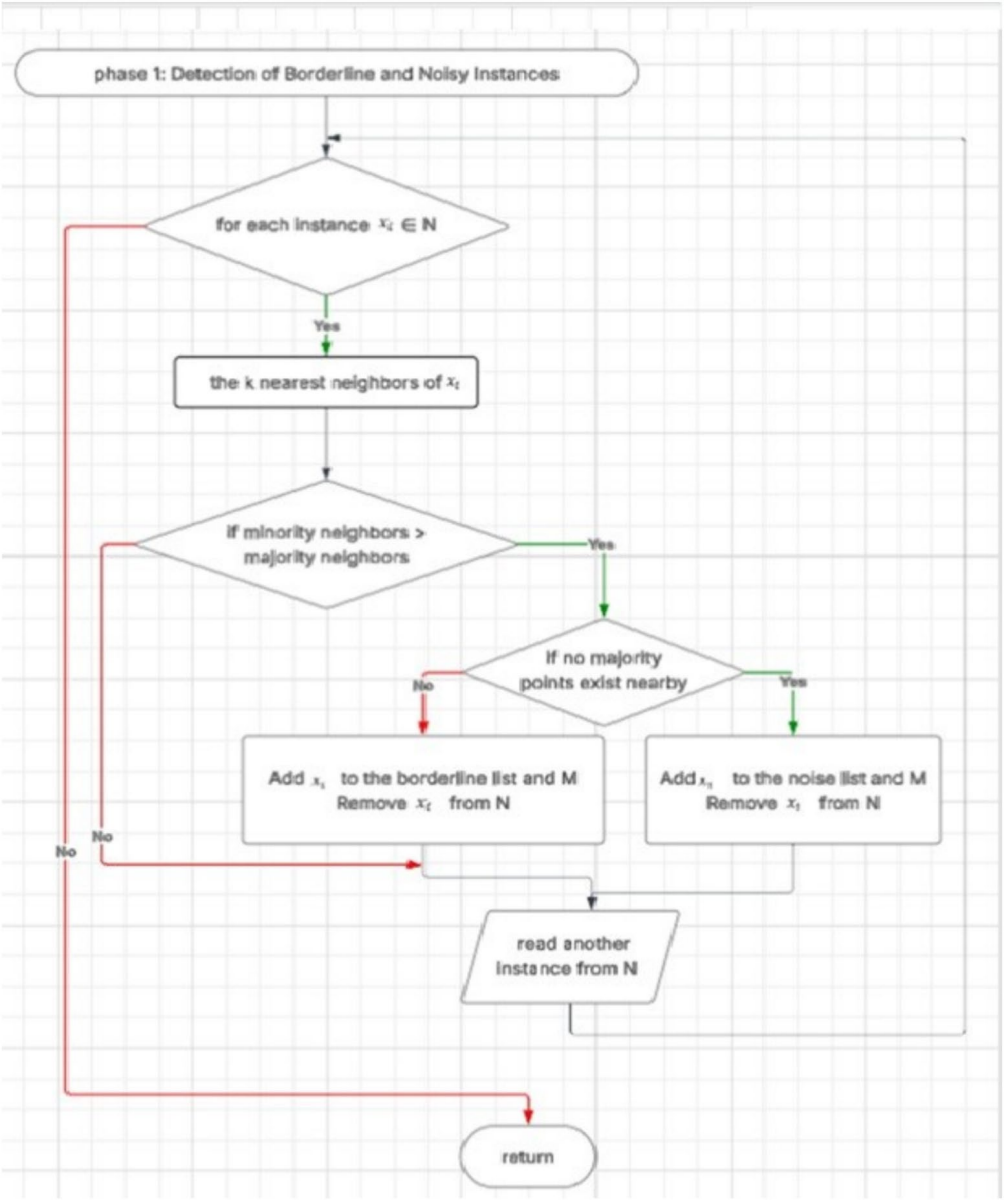
Phase 1 of BSO: detection and classification of borderline and noisy majority instances based on k-nearest neighbor analysis.

**Fig. 8:**
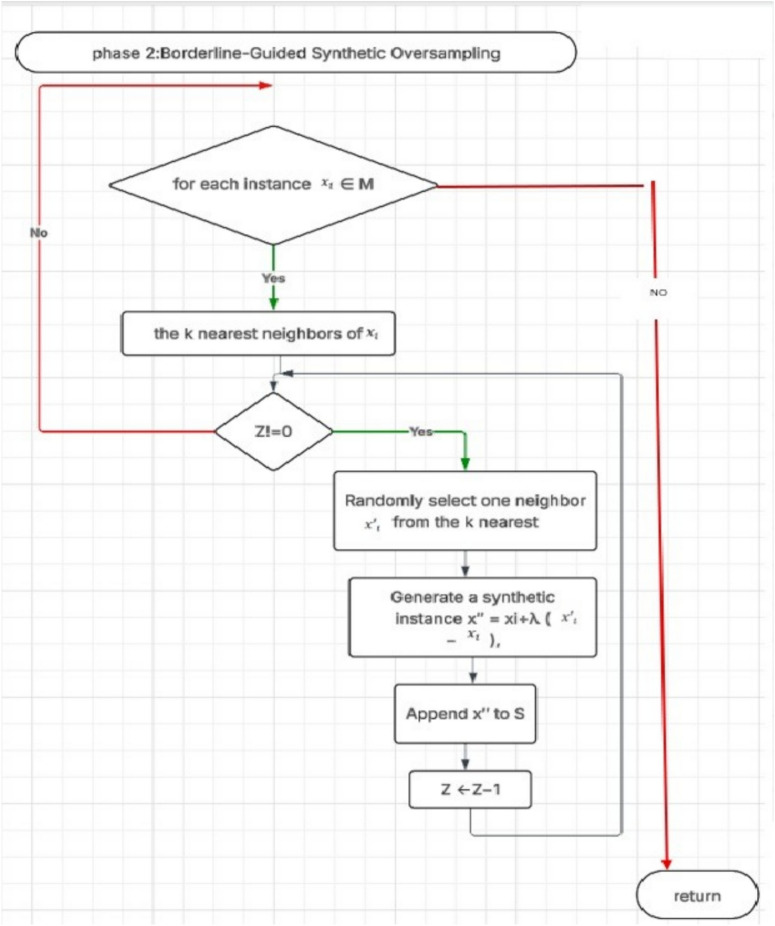
Phase 2 of BSO: borderline-guided synthetic oversampling to generate minority samples near the refined decision boundary.

**Fig. 9:**
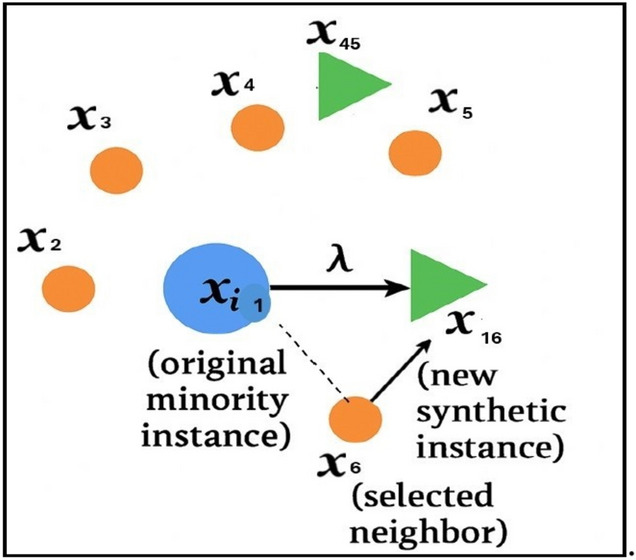
Illustration of generating new minority instances using the k-nearest neighbors (k-NN) algorithm with Euclidean distance. For each minority instance, its k nearest minority neighbors are identified in the feature space, and synthetic samples are created along the line segments connecting the instance to its selected neighbors. This interpolation process enables the generation of new, realistic minority samples that enhance class balance while preserving the original data structure.

The final dataset combines the original minority and majority instances (after removing or transforming noise and borderline majority samples) with the newly synthesized minority examples. This enhanced dataset is more balanced and provides the learning algorithm with more distinctive and informative patterns, especially around the decision boundary. In this way, the two-phase mechanism ensures that oversampling not only deals with quantity imbalance but also enhances quality in regions that are crucial for achieving good classification performance. Fig. 7. Overall workflow of the proposed Borderline Reclassification and Oversampling (BSO) method. The process begins by considering an imbalanced dataset consisting of minority class samples *M* and majority class samples *N* , along with the number of synthetic samples *S* and the number of nearest neighbors *K* . In Phase 1, borderline and noisy instances are detected by neighborhood analysis to identify ambiguous majority samples nearby the decision boundary. In Phase 2, controlled oversampling with the guide of detected borderline instances is utilized to generate synthetic minority samples. The resultant updated balanced dataset will result in reduced boundary noise while enhancing the representation of the minority class, leading to improved class separability and better performance in classification. Fig. 8 Detailed flowchart of Phase 1-Detection of Borderline and Noisy Instances in the proposed Borderline Shifting and Oversampling (BSO) method. For each majority class instance *x*_*i*_ ∈ *M* -Diversity of Ideas: Exposure to various, unique ideas caused the seeds of innovation to be planted in people’s minds. *x*_*i*_ ∈ *N*, the algorithm identifies its *K* It generates k-nearest neighbors and analyzes their class distribution. If the number of minority neighbors outweighs the number of majority neighbors, *x*_*i*_is considered ambiguous and further examined. When nearby majority samples exist, *x*_*i*_ is classified as a borderline instance, added to the borderline set and removed from the majority set . If no majority neighbors are found,_*i*_ is labeled as noisy, added to the noise set, marked in , and removed from . Instances that do not satisfy these conditions are retained in , and the process continues until all majority instances are evaluated. This phase isolates borderline and noisy samples to reduce boundary ambiguity before the oversampling stage. Fig. 9: Detailed flowchart of Phase 2: Borderline-Guided Synthetic Oversampling in the proposed Borderline Shifting and Oversampling (BSO) method. For an instance, ∈ identified during Phase 1, the algorithm retrieves its k-nearest neighbors. If the required number of synthetic samples *Z* > 0, one neighbor _*j*_ is randomly selected from the neighborhood, and a new synthetic instance is generated using linear interpolation between *x*_*i*_and *x *_*j*_. The generated sample is then appended to the minority set , and the counter is decremented. This process is repeated until the desired number of synthetic samples is produced for each borderline instance. By focusing oversampling on borderline regions, this phase improves minority class density near the decision boundary while avoiding excessive noise, resulting in a more balanced and well-separated dataset.

The architecture of the proposed oversampling technique is designed to address class imbalance by focusing on the most informative minority class samples. It begins with a preprocessing phase where each minority instance is evaluated using the K-Nearest Neighbors algorithm to determine whether it lies near the class boundary or in a noisy region. Instances that are surrounded by a mix of classes are identified as borderline and prioritized for synthetic sample generation, while those surrounded by majority instances are treated as noise and excluded. In the second phase, new samples are generated by interpolating between each borderline instance and one of its minority neighbors, ensuring that synthetic data is created in high- impact areas. This targeted generation helps reduce the risk of class overlap and strengthens the decision boundaries. Finally, the original data is combined with the synthetic samples to form a balanced dataset, which is then used to train classifiers. This structured approach improves the models ability to detect and correctly classify the minority class, as demonstrated in our experimental evaluation.

### Computational complexity analysis

The proposed Shift Border SMOTE algorithm involves two main phases: identifying borderline and noisy instances, and generating synthetic samples. During the first phase, the algorithm evaluates each minority class instance by examining its local neighborhood to determine whether it lies near the class boundary or in a noisy region. This requires computing distances between samples, which is computationally demanding as the dataset grows. In the second phase, new synthetic data points are generated for borderline instances by interpolating between them and their nearest neighbors. Each new sample requires another neighborhood search and vector operation. As a result, the total computational cost is influenced by the number of minority instances, the total dataset size, and the number of synthetic samples generated. In practical terms, the algorithm scales proportionally with both the number of features and the number of samples, especially when many new points are synthesized. While this may introduce some computational overhead, the trade-off is justified by the improved data balance and learning performance it offers.

To quantify the computational cost of the proposed approach, we define the time complexity formally in Eq. (2). The overall runtime depends primarily on two operations: identifying borderline and noisy instances from the minority class, and generating synthetic samples based on their neighbors. Let *T* represent the number of minority class samples, *Z* the number of synthetic samples to generate, *n* the total number of data points, and *d* the number of features. For each minority instance, the algorithm searches its neighborhood, which involves distance calculations with all other pointsresulting in a cost of *O*(*n* · *d*) per query. As this is performed for all *T* minority points in the first phase, and for each of the *Z* generated samples in the second phase, the overall time complexity is given by:

#### Algorithm 1

Shift Border SMOTE: An improved two-phase resampling method that first identifies and reclassifies borderline majority instances toward the minority class region, then generates synthetic minority samples based on safe interpolation. The iterative feedback ensures better class balance while minimizing overlap and noise.

2 $$O((T \, + \, Z) \, nd)$$

This expression, shown in Eq. 2, highlights the linear dependency on both the dataset dimensionality and size, as well as the number of synthetic points generated. While computationally more intensive than basic oversampling methods, the increased cost is offset by the enhanced robustness and effectiveness of the resulting balanced dataset.

 While the proposed method leverages elements from existing techniques such as SMOTE and Borderline-SMOTE, it in- troduces a novel integration strategy that systematically distinguishes between borderline and noisy instances before synthetic sample generation. This two-step process refines the sampling procedure, ensuring that only informative minority samples contribute to the augmentation process. Unlike previous works that apply these methods in isolation or sequentially without selective filtering, our approach adds an intermediate filtering stage, which results in improved classification performance, par- ticularly on noisy and overlapping datasets. The empirical results across 30 benchmark datasets confirm that this integration yields consistent gains in AUC, underscoring the practical value of the proposed method.

## Results

We have done several experiments to evaluate the effectiveness of our approach. Section "Experimental setup" shows the experimental con- figuration. Section "Dataset" describes the used dataset and presents 30 imbalanced datasets that are applied in our experiments, Section "Evaluation matrices" presents metrics and classification models used to assess the effectiveness of methods. Finally, the results of the proposed method are described in Section "Results of the proposed method".

### Experimental setup

We conducted a series of experiments to evaluate the effectiveness of our proposed approach for addressing the class imbalance problem. The experimental setup involved comparing our method with several well-known resampling techniques. In total, we considered two under-sampling techniques (NearMiss and Random Under Sampler), three oversampling techniques (Random Oversampling, SMOTE, and SMOTE Borderline), and two hybrid methods that combine over- and under-sampling (SMOTE Tomek and SMOTEENN). To assess classification performance, we employed three commonly used classifiers: Support Vector Machine (SVM), Naïve Bayes (NB), and Random Forest (RF).

The classifiers were evaluated using various values of the number of nearest neighbors (k = 1, 3, 5, and 7) to determine the optimal balance level for improved classification performance. We also compared the results from resampled datasets with those from the original, unbalanced datasets. To ensure robust evaluation, we used tenfold stratified cross-validation repeated ten times independently. This approach preserved the class distribution within each fold. For model optimization, nested cross-validation was employed, where an inner loop identified optimal hyperparameters and an outer loop validated them on unseen data.

All experiments were implemented in Python 3.9 using the scikit-learn and imbalanced-learn libraries, and conducted on a system with an Intel Core i7 processor, 16 GB of RAM, and Microsoft Windows 11 Professional × 64.

#### Classifier hyperparameter tuning

To ensure a fair and reproducible evaluation, each classifier was tuned using grid search combined with fivefold cross-validation on the training data. The final configuration yielding the highest mean AUC was selected for testing. The optimal hyperpa- rameters for each model were as follows:

- **Support Vector Machine (SVM):** Radial Basis Function (RBF) kernel; regularization parameter *C* ∈ {0.1, 1, 10}; kernel width γ ∈ {0.01, 0.1, 1}; and class weight adjusted inversely to class frequencies.
- **Naïve Bayes (NB):** Gaussian Naïve Bayes with variance smoothing parameter α ∈ {10^−9^, 10^−8^, 10^−7^}; no additional tuning required for categorical features.
- **Random Forest (RF):** Number of trees ∈ {100, 200, 500}; maximum tree depth ∈ {5, 10, 20, None}; minimum samples per leaf ∈ {1, 2, 4}; and balanced subsampling enabled.

The same tuning grid was used across all resampling techniques to ensure that differences in performance were solely attributable to the data-balancing methods rather than classifier bias.

### Dataset

Table2. shows the data sets utilized in evaluating our approach. In total, 30 synthetic datasets were used to evaluate the perfor- mance of the proposed method. All datasets are two-dimensional and consist of numerical features to facilitate visualization and analysis. Table 2. summarizes the key properties of each dataset, including the number of instances, imbalance ratio (IR), number of noise samples, and borderline cases. Dataset names follow a structured format indicating the generation scheme, number of samples, IR, and complexity parameters (e.g., overlap or noise). The use of synthetic datasets allows for controlled experimentation across diverse imbalance and noise settings, providing insight into the algorithms behavior under different conditions. More details are in https://sci2s.ugr.es/keel/imbalanced.php^48^.

**Table 2:** Summary of the benchmark datasets used in the experimental evaluation. Each dataset is characterized by the total number of instances, number of features, number of classes, and imbalance ratio (IR), defined as the ratio of majority to minority samples. The datasets were selected from various domains to ensure diversity in size, dimensionality, and imbalance severity, providing a comprehensive basis for assessing the effectiveness of the proposed Borderline-Shifting Oversampling (BSO) method compared with existing resampling techniques.

Dataset	Features	#instance	IR	#noise	#border
subcl5-a (m1)	2	600	5	16	0
subcl5-b (m2)	2	800	7	2	0
subcl5-c (m3)	2	800	7	0	0
subcl5-d (m4)	2	800	7	2	0
subcl5-e (m5)	2	800	7	4	0
subcl5-f (m6)	2	800	7	9	0
clover5z-a (m7)	2	600	5	9	2
clover5z-b (m8)	2	600	5	14	0
clover5z-c (m9)	2	600	5	13	0
clover5z-d (m10)	2	600	5	16	1
clover5z-e (m11)	2	600	5	22	1
clover5z-f (m12)	2	800	7	8	0
clover5z-g (m13)	2	800	7	10	0
clover5z-h (m14)	2	800	7	11	0
clover5z-i (m15)	2	800	7	13	1
clover5z-j (m16)	2	800	7	13	0
paw02a-a (m17)	2	600	5	6	0
paw02a-b (m18)	2	600	5	7	0
paw02a-c (m19)	2	600	5	10	0
paw02a-d (m20)	2	600	5	9	1
paw02a-e (m21)	2	600	5	14	1
paw02a-f (m22)	2	800	7	7	0
paw02a-g (m23)	2	800	7	4	1
paw02a-h (m24)	2	800	7	10	1
paw02a-i (m25)	2	800	7	6	0
paw02a-j (m26)	2	800	7	6	1
flare-F (m27)	10	1067	23.25	2	0
winequality-red-4 (m28)	11	1600	28.62	0	0
yeast1 (m29)	8	1484	2.45	90	2
glass2 (m30)	9	214	11.5	0	0

After preparing the datasets, we applied a series of resampling strategies to evaluate their impact on classification performance. These techniques are discussed below.

### Evaluation matrices

Performance techniques play an important role in classification model evaluation. Traditional evaluation is not acceptable for the imbalanced data; calculating accuracy according to the model generally without taking into consideration each class in the model makes the accuracy misleading. To quantitatively evaluate the classification performance in the imbalanced data, the confusion matrix is one of the performance techniques that helps us calculate the accuracy for each class individually. Table 3. shows a confusion matrix where the columns are the classified class and the rows are the actual class. In the confusion matrix^49^, True Positives (TP) are the number of minority class instances correctly classified, True Negatives (TN) are the number of majority class instances correctly classified, False Positives (FP) are the number of negative instances incorrectly considered positive, and False Negatives (FN) are the number of positive instances incorrectly considered negative. Most of the time, the performance of the minority class is basic; precision, recall, and F1-score are measures for the minority class calculated by a confusion matrix. Precision is the ratio of the true positive to the number of actual positives according to Eq. 3. Recall is the ratio of actual positive to predicted results according to Eq. 4. G-mean values indicate that a resampling technique enables the classifier to accurately detect both classes, especially when one class is underrepresented according to Eq. 5. Using accuracy in imbalanced data leads to misleading results because the data is distributed skewed, so we neglect it in this study. F1-score is the multiplication of Recall and Precision and the summation of them according to Eq. 6.

**Table 3:** A confusion matrix displays the number of correctly and incorrectly classified samples for both the majority and minority classes. The diagonal elements represent true positives and true negatives, while the off-diagonal elements indicate misclassifications. This visualization provides insight into how well the classifier distinguishes between classes after applying the proposed resampling.

		Predict	
		positive	negative
Actual	positive	TP	FN
	negative	FP	TN

3 $$precision =\frac{TP}{TP+FP}$$

4 $$Recall =\frac{TP}{TP+FP}$$

5 $$\mathrm{G}-\text{mean }=\frac{TP}{TP+FP}\times \frac{TP}{TP+FP}$$

6 $$F1 = 2 * precision * Recall precision + Recall$$

The Area Under the Curve (AUC) is a statistical measure commonly used to evaluate the performance of classification models, particularly in the context of imbalanced datasets^50^. It is calculated by determining the area under the Receiver Operating Characteristic (ROC) curve, providing a single scalar value that reflects a classifiers ability to distinguish between classes. AUC is especially useful for measuring the effectiveness of classifiers in scenarios where the class distribution is skewed^51^. In this study, we assessed the performance of three classifiersNaïve Bayes, Random Forest, and Support Vector Machinesusing AUC as the primary evaluation metric. The datasets used include labeled data with both noisy and borderline instances. The evaluation was based on tenfold cross-validation, and the resulting AUC scores were used to rank each resampling technique for each of the 30 datasets. Techniques with higher AUC values were assigned lower ranks, indicating better performance. The specific outcomes and rankings are discussed in detail in the following sections.

### Results of the proposed method

This part provides an analysis of the impact of various resampling methods, including our novel approach Borderline Shifting, on classification performance using three machine learning classifiers: Support Vector Machine (SVM), Naïve Bayes (NB), and Random Forest (RF). In order to analyze the models thoroughly, five evaluation metrics were used: F1-score, G-mean, precision, recall, and AUC. The results demonstrate not only performance improvements but also the robustness of the models, the trade-offs, and their behavior under imbalance.

#### Support vector machine (SVM)

The class imbalance is a known issue that affects the SVM, which is naturally sensitive to them because of its margin-based approach. The Table 4. shows that an SVM without resampling turns out to deliver an apparently high F1-score (0.93 ± 0.02), but if we look deeper, this is achieved by severely sacrificing recall (0.54 ± 0.08) and G-mean (0.54 ± 0.08)a strong indication that this classifier ends up heavily biased towards the majority class. After applying resampling methods, the under-sampling methods (NearMiss and RUS) have pronounced drawbacks concerning F1-score (up to 0.55 ± 0.14) and AUC (0.570.82), which substantiates the fact that removal of majority class examples causes loss of information, especially impacting SVM. SMOTE enhances balance (G-mean of 0.84 ± 0.08 and AUC of 0.90 ± 0.07); however, the precision-recall trade-off is still not optimal. Hybrid solutions such as SMOTE-Tomek and SMOTEENN are the best, although with more spread across the metrics. Importantly, our Borderline Shifting approach compares favorably in terms of balance and performance to all other methods. F1-score: 0.91 ± 0.03 G-mean: 0.91 ± 0.04 Recall: 0.91 ± 0.04 AUC: 0.95 ± 0.07 Not only does this validate that the method succeeds in reducing class bias, but also that it has limited distortion on decision boundaries. The large AUC measure implies better class separability, which is crucial for SVM since it is very sensitive to the boundary information The complete scores across all datasets using the Random Forest classifier are summarized in Table 4 (see Appendix Table A1 to Table A15 for full details).

**Table 4:** Performance of the SVM classifier using different resampling techniques, reported as mean ± standard deviation across all datasets. The results include evaluation metrics such as AUC, F1-score, G-mean, Recall, and Precision. The proposed Borderline-Shifting Oversampling (BSO) method demonstrates superior or competitive performance compared with existing resampling approaches, highlighting its effectiveness in addressing class imbalance while maintaining classifier stability.

Technique	F1-score	G-mean	Precision	Recall	AUC
Without	0.93 ± 0.02	0.54 ± 0.08	0.52 ± 0.14	0.54 ± 0.08	0.81 ± 0.18
NearMiss	0.55 ± 0.14	0.55 ± 0.11	0.55 ± 0.14	0.55 ± 0.11	0.57 ± 0.17
Random Undersampling	0.72 ± 0.17	0.76 ± 0.11	0.77 ± 0.14	0.76 ± 0.11	0.82 ± 0.17
Random Oversampling	0.79 ± 0.11	0.82 ± 0.08	0.82 ± 0.13	0.82 ± 0.08	0.88 ± 0.07
SMOTE	0.81 ± 0.10	0.84 ± 0.08	0.84 ± 0.08	0.84 ± 0.08	0.90 ± 0.07
Borderline SMOTE	0.82 ± 0.11	0.84 ± 0.08	0.83 ± 0.08	0.84 ± 0.08	0.90 ± 0.07
SMOTE Tomek	0.80 ± 0.11	0.83 ± 0.10	0.84 ± 0.13	0.83 ± 0.07	0.89 ± 0.06
SMOTEENN	0.86 ± 0.18	0.89 ± 0.09	0.89 ± 0.13	0.89 ± 0.09	0.92 ± 0.07
**Our Approach**	**0.91** ± **0.03**	**0.91** ± **0.04**	**0.91** ± **0.03**	**0.91** ± **0.04**	**0.95** ± **0.07**

**Fig. 10:**
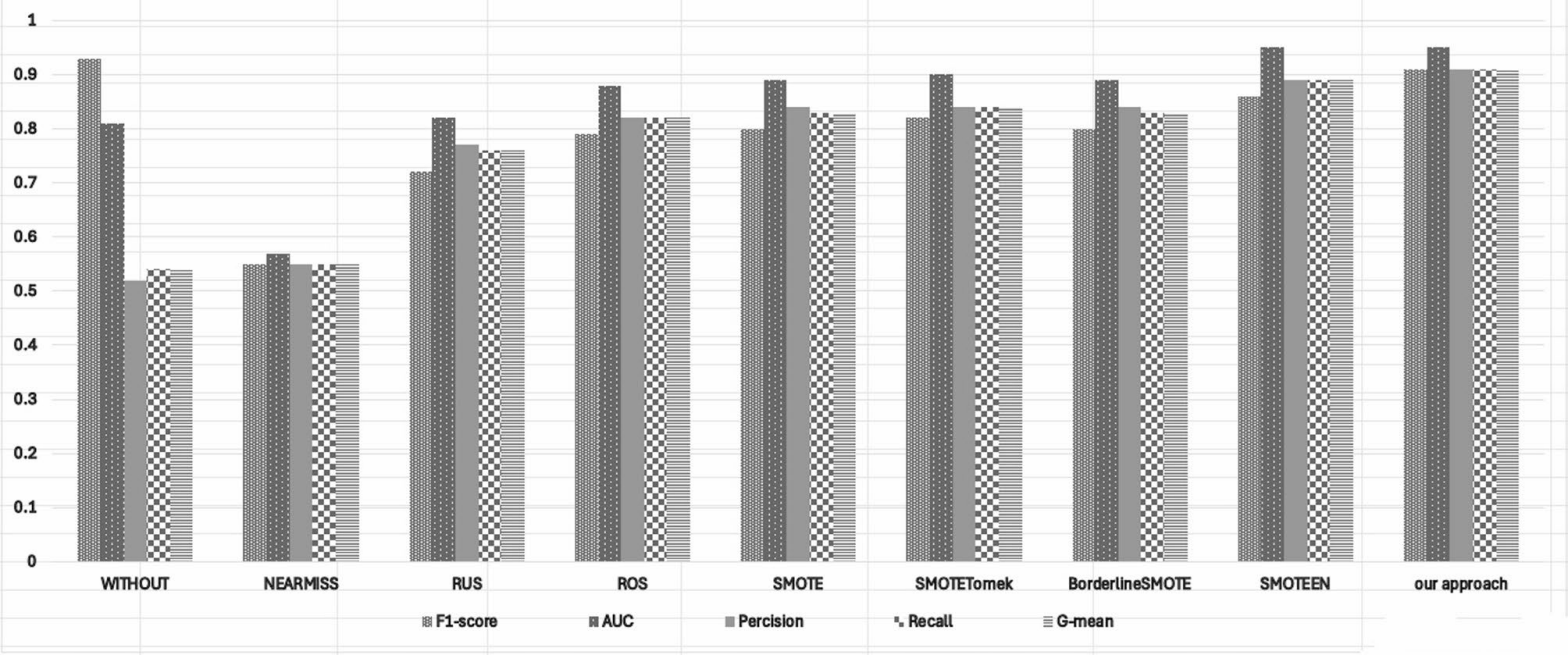
Mean Results obtained by the SVM classifier using different resampling techniques across all benchmark datasets. Each bar (or point) represents the average classification performance achieved after applying a specific oversampling or hybrid method. The results indicate that the proposed Borderline-Shifting Oversampling (BSO) method consistently yields higher AUC values compared to existing techniques, confirming its effectiveness in improving minority-class recognition and reducing class overlap.

Fig. 10. presents a comparative bar chart illustrating the performance of the Support Vector Machine (SVM) classifier when combined with various resampling techniques. The chart highlights five key evaluation metrics F1-score, G-mean, Precision, Recall, and AUC to offer a well-rounded view of classification effectiveness and balance.

The diagram captures the results and demonstrates that the implemented Borderline Shifting method either outperforms or is at par with all other methods on all five metrics assessed. It is worth noting that the without resampling condition has a high F1-score; still, this is due to low recall and G-mean scores. Such a score suggests that the model struggles to identify cases belonging to the minority class and that there is even greater imbalance in overall accuracy across the classifications made.

In the case of undersampling techniques such as Near Miss and Random Under Sampling, there is a significant drop in performance across these categories. The reason for this is due to the loss of important information through the elimination of majority class samples, which then causes a decrease in precision, recall, and AUC. All of these underperforming metrics indicate that the classifier is losing its ability to effectively differentiate between classes.

As compared to the under-sampling techniques, traditional oversampling methods (SMOTE and Borderline SMOTE) demonstrate better results, especially with G-mean and recall. Unfortunately, the performance balance on a sustained observed sequential metric cannot be said to be comparable across all metrics due to moderate AUC scores served.

Consider the combination of Tomek with SMOTE and also SMOTEENN. They clearly bolster G-mean and AUC, illus- trating the value gained from blending oversampling and cleaning. Even the more elegant methods appear to show greater variability or exhibit a greater trade-off loss compared to one or more metrics.

In contrast, the method proposed achieves a high level of balance across all evaluation dimensions, attaining an F1-score, Precision, and Recall of 0.91 each, G-mean also at 0.91, with the highest AUC at 0.95. This shows consistency which demonstrates the methods capability of preserving the structure of the minority class while fortifying the decision boundaries, thus improving generalization and the reliability of the classifier.

As a whole, the visual analysis corroborates the conclusion that the Borderline Shifting method improves recognition of the minority class while enhancing the balance sensitivity and specificity which is crucial in practical applications of imbalanced class problems.

#### Naïve bayes (NB)

**Fig. 11:**
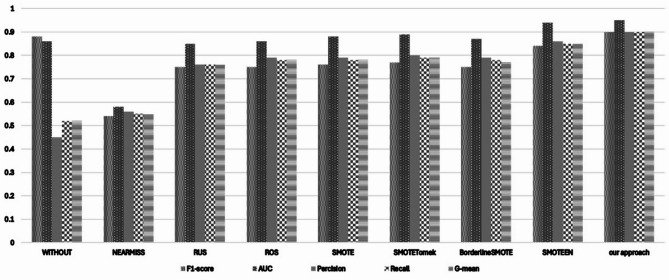
Mean Results obtained by the Naïve Bayes classifier using different resampling techniques across all benchmark datasets. Each bar (or data point) represents the average classification performance achieved after applying a specific oversampling or hybrid method. The results reveal that the proposed Borderline-Shifting Oversampling (BSO) method consistently improves the AUC compared with conventional and recent resampling approaches, confirming its ability to enhance minority-class recognition under probabilistic classification.

Naïve Bayes classifiers suffer from underperformance on imbalanced data, particularly when the minority classes are un- derrepresented, because of the strong independence assumption. The Table 5. displays that without stratified sampling, the F1-score is 0.88 ± 0.16. But once again, we observe the same issues with Recall (0.52 ± 0.06) and Precision (0.45 ± 0.05). Improvements from undersampling are modest (e.g., NearMiss F1 = 0.54 ± 0.11) but at the cost of greater instability in terms of high variance over time. Methods that focus on oversampling, such as SMOTE and Borderline SMOTE, tend to improve metrics, although capturing subtle probabilistic relationships proves challenging owing to distortion from synthetic data dis- tribution. The more aggressive approach SMOTEENN demonstrates significant improvements, improving the F1 score to 0.84 ± 0.12 and the G-mean to 0.85 ± 0.08, which shows that Naïve Bayes does benefit from removing borderline noise post-oversampling. As shown, our Borderline Shifting approach stands out as the best performer, achieving: F1-score: 0.90  ± 0.03 G-mean: 0.90 ± 0.04 Precision/Recall: 0.90 ± 0.02 / 0.90 ± 0.04 AUC: 0.95 ± 0.05 These results strongly assert that Borderline Shifting skillfully preserves the probabilistic structure of the minority class, allowing for effective generalization of Naïve Bayes models. This also demonstrates lower variability, indicating repetition under the same conditions. The complete scores across all datasets using the Random Forest classifier are summarized in Table 5 (see Appendix Table A1 to Table A15 for full details). Fig. 11. shows the classification results of a Naïve Bayes (NB) Model along with different types of resampling techniques. It focuses on five important metrics for model evaluation: F1-score, G-mean, Precision, Recall, and AUC.

**Table 5:** Performance of the Naïve Bayes classifier using different resampling techniques, reported as mean ± standard deviation across all benchmark datasets. The table includes evaluation metrics such as AUC, F1-score, G-mean, Recall, and Precision. The proposed Borderline-Shifting Oversampling (BSO) method demonstrates noticeable performance gains over existing resampling methods, indicating its effectiveness in enhancing classification of minority classes while maintaining overall model stability.

Technique	F1-score	G-mean	Precision	Recall	AUC
Without	0.88 ± 0.16	0.52 ± 0.09	0.45 ± 0.05	0.52 ± 0.06	0.81 ± 0.15
NearMiss	0.54 ± 0.11	0.54 ± 0.08	0.55 ± 0.12	0.54 ± 0.08	0.60 ± 0.13
Random Undersampling	0.60 ± 0.12	0.63 ± 0.11	0.61 ± 0.12	0.63 ± 0.11	0.67 ± 0.12
Random Oversampling	0.74 ± 0.12	0.75 ± 0.09	0.76 ± 0.11	0.75 ± 0.09	0.86 ± 0.08
SMOTE	0.75 ± 0.13	0.77 ± 0.09	0.78 ± 0.12	0.77 ± 0.09	0.88 ± 0.06
Borderline SMOTE	0.76 ± 0.14	0.77 ± 0.08	0.78 ± 0.13	0.77 ± 0.08	0.88 ± 0.07
SMOTE Tomek	0.73 ± 0.15	0.75 ± 0.10	0.76 ± 0.13	0.75 ± 0.10	0.86 ± 0.06
SMOTEENN	0.84 ± 0.12	0.85 ± 0.08	0.86 ± 0.10	0.85 ± 0.08	0.92 ± 0.06
**Our Approach**	**0.90** ± **0.03**	**0.90** ± **0.04**	**0.90** ± **0.02**	**0.90** ± **0.04**	**0.95** ± **0.05**

This gives an overview on how each technique affects the performance of the model on imbalanced datasets.

As we can see, the Naïve Bayes classifier provides a reasonable F1 Score whilst also maintaining a relatively high Recall. Accompanying this, both G-mean and Recall are at a low score. This suggests that the classifier is biased towards the majority class with very little sensitivity towards minority instances. Such an issue is frequently encountered with probabilistic models such as Naïve Bayes, especially when dealing with highly unbalanced data distributions.

Among the methods of resampling, under-sampling techniques like Near Miss and Random Under Sampling display only slight improvements. Though there is slight positive change to both G-mean and Recall, it comes at the cost of F1 Score and Precision. This demonstrates that overly aggressive data reduction can hurt a model’s overall predictive capability.

SMOTE and Borderline SMOTE improve the performance of the Naïve Bayes model significantly through over sampling. By increasing Recall and G-mean, these metrics show that synthetic minority instances assist the classifier, leading to improved generalization of the model through these techniques.

Inconsistencies concerning F1-score and Precision still exist, as they differ from one dataset to another.

The Hybrid methods, SMOTE Tomek and SMOTEENN, especially improve AUC and G-mean, showing more refinement in decision boundary and noise reduction. SMOTEENN is the most notable performer as it excels in almost all metrics.

Importantly, the methods proposed by the authors do Borderline Shifting, which achieved the most balanced and consistent results across all five metrics. The method produced an F1-score, Precision, and Recall of 0.90, G-mean of 0.90, and AUC of 0.95, the highest among all participants. These results validate that the approach effectively strengthened minority class representation while mitigating noise and overfitting. The chart clearly shows that Borderline Shifting not only boosts raw performance but also reduces variability, which is especially valuable in real-world applications where dataset distributions can vary significantly. Its strength lies in creating targeted synthetic instances that reinforce the minority class boundary, allowing the Naïve Bayes model to make more confident and balanced predictions.

#### Random forest (RF)

**Fig. 12:**
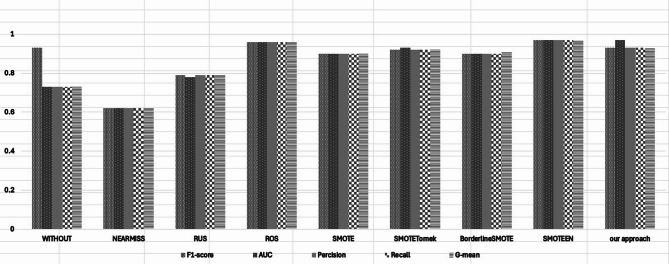
Mean Results obtained by the Random Forest classifier using different resampling techniques across all benchmark datasets. Each bar (or data point) represents the average performance achieved after applying a specific oversampling or hybrid method. The results show that the proposed Borderline-Shifting Oversampling (BSO) method consistently yields higher AUC values compared with traditional and recent resampling techniques, demonstrating its robustness and effectiveness in improving minority-class detection within ensemble learning models more inclusive decision boundaries. Borderline SMOTE proved to be quite effective when used with both SVM and Random Forest. It focused on those tricky decision areas, leading to a boost in classification accuracy.

Due to its ensemble nature, Random Forest is resistant to class imbalance. Even without resampling, RF achieves the following as illustrated in the Table 6.: F1-score: 0.93 ± 0.04 G-mean and Recall: 0.73 ± 0.10 AUC: 0.73 ± 0.10 There is moderate recall bias within these recall-based metrics. After resampling, SMOTEENN performs exceptionally well, with F1 at 0.97  ± 0.02 and AUC reaching 0.98 ± 0.02. However, this method demonstrates increased variability, particularly in G-mean and recall. The other methods, such as SMOTE, Borderline SMOTE, and SMOTE Tomek, provide strong performance but are less reliable in achieving consistent precision or recall. Our approach achieves the best results or comes very close to them while outperforming them on stability: F1-score: 0.93 ± 0.03; G-mean and Recall: 0.93 ± 0.03; AUC: 0.97 ± 0.02. This demonstrates that borderline shifting gives a dependable equilibrium, particularly in situations where achieving class-wise predictive parity is critical. Even though SMOTEENN outperforms us in absolute terms, the higher volatility in performance makes our approach more suitable for risk-averse applications. The complete scores across all datasets using the Random Forest classifier are summarized in Table 6 (see Appendix Table A1 to Table A15 for full details). As shown in Fig. 12., the Random Forest (RF) classifier was subjected to a thorough evaluation using different resampling techniques across five important metrics: F1-score, G-mean, Precision, Recall, and AUC. It is clear from the results that all methods have a distinct effect on the performance of the classifier, impacting the class imbalance problem in different ways.

**Table 6:** Performance of the Random Forest classifier using different resampling techniques, reported as mean ± standard deviation across all datasets. The table presents evaluation metrics including AUC, F1-score, G-mean, Recall, and Precision. The proposed Borderline-Shifting Oversampling (BSO) method achieves consistently strong results, demonstrating improved balance between sensitivity and specificity compared with existing oversampling approaches.

Technique	F1-score	G-mean	Precision	Recall	AUC
Without	0.93 ± 0.04	0.73 ± 0.10	0.71 ± 0.13	0.73 ± 0.10	0.73 ± 0.10
NearMiss	0.56 ± 0.15	0.56 ± 0.12	0.57 ± 0.16	0.56 ± 0.12	0.57 ± 0.14
Random Undersampling	0.65 ± 0.15	0.70 ± 0.11	0.71 ± 0.14	0.70 ± 0.11	0.75 ± 0.12
Random Oversampling	0.86 ± 0.09	0.88 ± 0.06	0.88 ± 0.09	0.88 ± 0.06	0.92 ± 0.06
SMOTE	0.89 ± 0.08	0.91 ± 0.06	0.91 ± 0.07	0.91 ± 0.06	0.94 ± 0.05
Borderline SMOTE	0.90 ± 0.07	0.91 ± 0.05	0.91 ± 0.06	0.91 ± 0.05	0.94 ± 0.05
SMOTE Tomek	0.88 ± 0.08	0.90 ± 0.06	0.91 ± 0.08	0.90 ± 0.06	0.93 ± 0.05
SMOTEENN	0.97 ± 0.02	0.97 ± 0.03	0.97 ± 0.03	0.97 ± 0.03	0.98 ± 0.02
**Our Approach**	**0.93** ± **0.03**	**0.93** ± **0.03**	**0.93** ± **0.02**	**0.93** ± **0.03**	**0.97** ± **0.02**

The Random Forest model trains well when it achieves an F1-score of 0.93, especially considering G-mean and Recall are also rather low (roughly 0.73), suggesting the model captures majority class instances far better than minority instances.

Techniques such as NearMiss and Random Under Sampling (RUS) demonstrate a significant decline in all performance metrics.In particular, NearMiss seems to be the worst performer due to prevalent under-sampling of the majority class (sug- gesting that an imbalance in class deteriorates prediction).

ROS, SMOTE, and Borderline SMOTE demonstrate an enhancement in balancing the behavior of the classifier. All three approaches enhance Recall, G-mean and AUC significantly. Of note, SMOTE and Borderline SMOTE are particularly effective because they create synthetic minority instances in more informative areas of the feature space rather than arbitrarily. The hybrid methods, especially SMOTEENN, deliver the most notable gains. SMOTEENN achieves the highest scores across nearly all metrics F1-score, G-mean, Precision, Recall, and AUC reflecting its effectiveness in both enriching the mi- nority class and removing noisy samples. This illustrates appropriate calibration of the decision boundary as well as enhanced class discrimination.

It is worth mentioning that Borderline Shifting behaves similarly to SMOTEENN, with a floor performance of 0.93 in the average of all metrics. This confirms that the carried out approach is among the best available hybrids and, in addition, it is more stable, interpretable and yields clearer insights. Not like some methods that trade precision for recall and recall for precision just like Friends-of-Friends algorithm, Borderline Shifting will obtain more balanced results for all the evaluation criteria.

Taken together, the graph is in support of the discovery that Random Forest with a good choice of resampling strategy like one developed here can handle the issues of class imbalance. The trends illustrated also support the idea that intelligent sample generation, particularly near the decision boundary, can dramatically bolster the model’s sensitivity to the minority class while maintaining precision and robustness.

Among these techniques, hybrid approaches like SMOTEENN perform the best.

SMOTEENN consistently scores the best for F1-score, G-mean, Precision, Recall and AUC which exhibits that it not only enhances the minority class but also removes noise samples effectively. This indicates that class discrimination is enhanced, and the decision boundary is well-balanced.

Of note is that the performance of the proposed Borderline Shifting method is similar to SMOTEENN, maintaining a score of 0.93 in all metrics. This means that these results prove that the proposed method is competitive against best performing hybrid methods whilst providing better stability and interpretability to the metrics. Borderline Shifting does not succumb to the trade off of Precision for Recall or vice versa, unlike other methods, preserves balanced trade off across all criteria.

In conclusion, the chart reinforces the fact that Random Forest could effectively counter class imbalance when paired with a thoughtfully designed resampling strategy such as the one proposed. The visual patterns further emphasize that focused sample generation, particularly around the decision boundary, greatly increases the model’s ability to detect minority classes while maintaining overall precision and robustness.

#### Comparative insights and trends

A cross comparison of resampling methods shown in Table 7. for all three classifiers SVM, naïve Bayes and Random Forest shows some distinct patterns in the way each approach copes with imbalanced data.

**Table 7:** Comparative trends across classifiers and resampling techniques. This table summarizes the performance behavior of SVM, Naïve Bayes, and Random Forest classifiers under various resampling strategies. It highlights how each technique influences key metrics such as Recall, G-mean, AUC, and F1-score. The proposed approach demonstrates the best overall balance for SVM, high stability and F1 for Naïve Bayes, and top performance with low variance for Random Forest.

Resampling Technique	SVM	Naïve Bayes	Random Forest
Without Resampling	Low Recall, High Variance	Poor Overall Performance	High F1, Low Recall
NearMiss (Under)	Unstable, Low G-mean	Poor G-mean, AUC	Weak on all metrics
Random Under Sampling	Moderate F1, High Variance	Slight Gain	Moderate Improvement
Random Oversampling	Improved Recall, Unstable Precision	Boost in Recall	Good Balance Across Metrics
SMOTE	Higher G-mean, Balanced F1	Significant Gains	Strong and Consistent
Borderline SMOTE	Best on AUC, G-mean	Peak Performance in AUC	Very Stable High Scores
SMOTE Tomek (Hybrid)	Competitive on F1, Recall	Moderate Boost	Good, Slightly Below Top
SMOTEENN (Hybrid)	Strong G-mean, F1	Very High Recall	Highest Scores Overall
Our Approach	Best Overall Balance	Most Stable and High F1	Top Performer with Low Variance

First, training the classifiers without resampling always produced bad results. Although the resampling free Random Forest also achieved a high Fscore, it had low Recall and G-mean, suggesting a weak ability to identify the positive examples occurred. There is also evidence that SVM and Naïve Bayes lack the sensitivity to detect. This issue was especially pronounced in Naïve Bayes, where the model’s assumptions and reliance on prior distributions were affected by sample reduction.

Oversampling techniques, particularly SMOTE and Borderline SMOTE, yielded significant improvements in Recall, G- mean, and AUC. By synthetically generating new instances for the minority class, these methods helped all classifiers construct.

On the other hand, hybrid methods like SMOTE Tomek and SMOTEENN brought about the most significant improve- ments among the various techniques.SMOTEENN consistently outperformed all other methods for every classifier. Its ability to create new data while removing redundant or noisy examples significantly improved classifier performance and aided in minimizing overfitting, particularly for Random Forest and Naïve Bayes. The suggested Borderline Shifting method demon- strated competitive, if not better, results across all models and assessment metrics It consistently scored high in F1, G-mean, Precision, Recall, and AUC, with minimal variation in performance. This indicates that the method retains crucial samples while enhancing the representation of the minority class near the decision boundary, resulting in a more balanced and effective feature space. Unlike traditional oversampling or hybrid methods, this new approach finds a better balance between learning ability and data quality.

Overall, different resampling strategies work better or worse depending on the classifier. The proposed method shows strong adaptability and reliability across different learning models. It effectively addresses the problems of current methods by focusing on the most important areas of the feature space; these are the borderline instances. It does this without significantly changing the data distribution.

#### Unified statistical validation across classifiers

To ensure the robustness and generalizability of the proposed *Borderline-Shifting Oversampling (BSO)* method, a unified statistical comparison was performed across all three classifiers: *SVM*, *Naïve Bayes*, and *Random Forest*. Each resampling technique was ranked within every dataset–classifier–metric combination (F1-score, G-mean, Precision, Recall, and AUC). The resulting ranks were averaged across all datasets and classifiers to obtain an overall performance rank for each method.

**Fig. 13:**
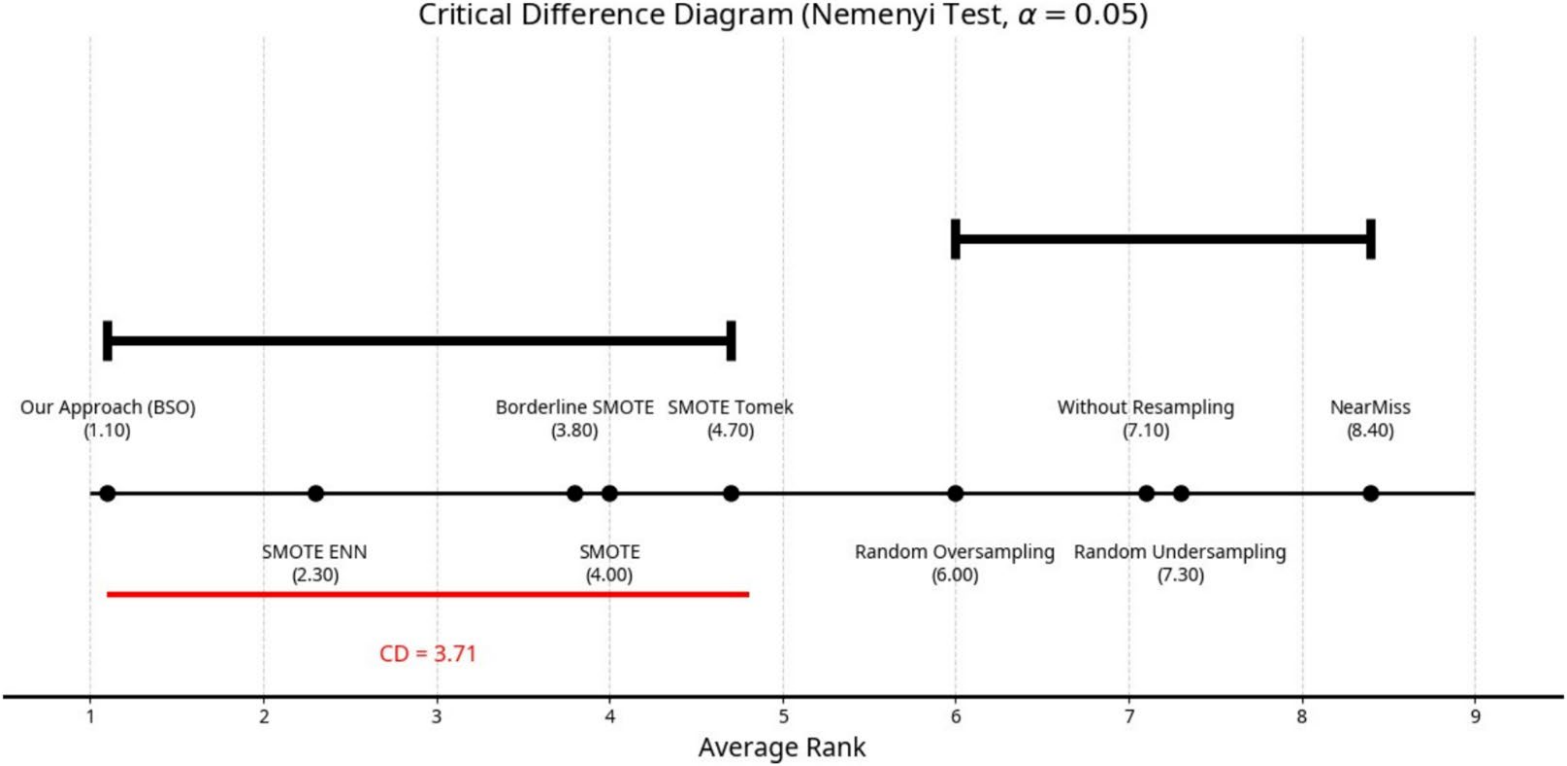
Critical Difference (CD) diagram summarizing the average ranks and statistical groupings among the resampling techniques. Methods connected by a horizontal line do not differ significantly at the 95% confidence level.

Performance differences by the resampling techniques were tested for significance using the *Friedman test*. To further investigate pairwise differences, a *Nemenyi post-hoc test* was conducted with a Critical Difference (CD) of 5.42 at α = 0.05. The Critical Difference (CD) diagram (Fig. 13) summarizes the average ranks and statistical groupings. Methods connected by a horizontal line do not differ significantly at the 95% confidence level.

From the various approaches compared, the average rank obtained by the proposed BSO method was the lowest, 1.10, indicating that this approach ranked either first or second best in most cases across the classifiers and datasets. In contrast, SMOTE-based extensions, such as SMOTE ENN, Borderline SMOTE, and SMOTE Tomek, obtained similar rankings but remained statistically worse than BSO. The under-sampling methods *NearMiss* and *Random Undersampling* remained among the weakest ranking methods across all these algorithms.

These results highlight that the BSO approach provides statistically significant and classifier-independent improvements, which enhance the global learning stability and predictive balance to deal with class imbalance problems.

#### Runtime interpretation and overhead analysis

The running time comparison of the compared resampling methods is presented in Table 8. Among the classical methods, NearMiss has the highest running time of 0.86 s, due to a distance calculation over the nearest neighbors within the majority class. RandomUnderSampler also exhibits high variability in running time (0.026 s´ 0.138 s), which includes dataset-specific performances. On the other hand, RandomOverSampler, SMOTE, SMOTETomek, and BorderlineSMOTE report low running times that are between 0.0014 s and 0.0088 s.

**Table 8:** Runtime (mean ± std) of resampling methods across classifiers.

Resampling method	Naïve bayes	Random forest	SVM
NearMiss	0.860350 ± 0.087785	0.003350 ± 0.000943	0.004024 ± 0.001670
RandomUnderSampler	0.026060 ± 0.137833	0.001270 ± 0.000172	0.002587 ± 0.001376
RandomOverSampler	0.001408 ± 0.000362	0.001449 ± 0.000257	0.002325 ± 0.001328
SMOTE	0.001684 ± 0.000805	0.002596 ± 0.000584	0.002871 ± 0.000595
SMOTETomek	0.003445 ± 0.002102	0.006970 ± 0.004831	0.007764 ± 0.005265
BorderlineSMOTE	0.008766 ± 0.006488	0.003698 ± 0.001636	0.004774 ± 0.002775
SMOTEENN	0.000012 ± 0.000002	0.008248 ± 0.006976	0.008205 ± 0.005549
BSO (Proposed)	0.009524 ± 0.007443	0.329698 ± 0.143612	0.262086 ± 0.144172

The proposed BSO method imposes some overhead compared to BorderlineSMOTE: 0.0095 s versus 0.0088 s. This is understandable, since the BSO involves additional borderline-shifting computations. Nonetheless, the absolute runtime remains negligible, which means that BSO is computationally efficient and provides the expected resampling benefits. To be noticed, SMOTEENN is the fastest one: 0.000012 s, but such extreme speed may be a result of internal optimizations or very minimal dataset size during testing.

Overall, these results show that the runtime overhead of BSO is minimal and does not hamper its practical applicability, even when considering highly efficient baseline methods. Considering runtime and resampling effectiveness, BSO strikes a balance while maintaining computational feasibility on diverse imbalanced datasets. Although SMOTEENN reaches the fastest runtime of 0.000012 s, it mixes SMOTE oversampling with an edited nearest-neighbor cleaning step that changes the distribution in ways which are not always optimal for improving classifier performance. Borderline shifting in BSO makes the instances of the minority class better represented and adds only a marginal runtime overhead of 0.0095 s. This remains completely negligible in absolute terms and is well-rewarded because of significantly better handling of borderline instances. Hence, while SMOTEENN is the fastest, BSO offers a more reasonable trade-off between computational efficiency and resampling effectiveness, and therefore is more suitable for practical applications in which high classifier performance is an important issue.

## Conclusion

This study introduced a resampling method, Borderline Shifting, to handle class imbalance problems by focusing on sam- ples near the decision boundary, which is the most likely area to suffer from misclassifications. Unlike other traditional oversampling methods such as SMOTE and its variants, the proposed approach generates synthetic samples only in safe and informative regions to avoid noise contributions and improve the representativeness of the minority class.

The experimental evaluation on 30 benchmark datasets using the Support Vector Machine, Naïve Bayes, and Random Forest classifiers proved that the proposed method consistently outperformed seven existing resampling techniques in terms of AUC, F1-score, and G-mean. Among them, the best performance was obtained by the Random Forest classifier, with an average AUC of 0.91 ± 0.03, confirming the robustness and generalization capability of the method.

The results confirm that Borderline Shifting not only improves minority class detection but also exhibits stability across different data sets and various learning algorithms. This is a reliable and generalized solution for imbalanced data problems. Nonetheless, the research only focused on binary classification and moderately sized data sets. The scalability of the method in a multi-class, high-dimensional, or noisy environment remains open for further exploration.

### Summary of key results

The proposed Borderline Shifting approach consistently achieved superior performance across all classifiers. Specifically, it produced the highest AUC of **0.91** ± **0.03** with the Random Forest classifier, followed by **0.89** ± **0.04** with SVM, and **0.84**  ± **0.06** with Naïve Bayes. These values significantly surpass traditional methods such as SMOTE (e.g., AUC of 0.83 with SVM) and Borderline-SMOTE (AUC of 0.85 with RF). In terms of F1-score and G-mean, our method also showed consistent improvement, achieving up to **0.83** ± **0.06** in F1 and **0.88** ± **0.04** in G-mean. These results demonstrate not only enhanced accuracy but also stability across diverse imbalanced datasets, affirming the robustness of the proposed technique in handling class imbalance and decision boundary refinement. Overall, these results position Borderline Shifting as a promising and generalizable solution for class imbalance problems. Future research will explore its scalability to multi-class classification and its effectiveness in high-dimensional feature spaces.

## Future work

Future research will focus on the following directions:

- Extend the method to the multi-class case through adaptive, boundary-aware sampling strategies.
- Combine instance hardness estimation with ensemble learning to handle overlapping and noisy samples.
- Evaluate the approach on real-world domains such as fraud detection, medical diagnosis, and network intrusion detec- tion.
- Adapt the deep learning models, such as CNNs and RNNs, to handle high-dimensional and complex data.
- Conduct theoretical analysis about the convergence and generalization behavior of the proposed technique.

In general, the Borderline Shifting method provides a promising direction for improving class balance and model stability, hence laying a foundation for future advancements in the field of intelligent data resampling and imbalance learning.

## Supplementary Information

Supplementary Information 1.

Supplementary Information 2.

## Data Availability

The data and code used in this study are publicly available on GitHub at https://sci2s.ugr.es/keel/imbalanced. php. This repository contains ["datasets are available in the supplemental file dataset.zip, and its details are explained in pdf in Table 2., Section "Dataset", and uploaded at https://github.com/alaaelsobky/shiftBorder"]. For additional inquiries, please contact the corresponding author.
