# Supplementary material for: An approach for handling imbalanced datasets using borderline shifting
Source: Sci Rep. 2026 Mar 4;16:8264. doi: 10.1038/s41598-026-39118-x (PMC12963434; doi:10.1038/s41598-026-39118-x)
Supplement: Supplementary file 1 — Supplementary Information 1. [file 41598_2026_39118_MOESM1_ESM.pdf]

A My Appendix

Table A1. Precision scores of SVM classifier across datasets using different resampling methods

Dataset	WITHOUT	NEARMISS	RUS	ROS	SMOTE	SMOTETomek	BorderlineSMOTE	SMOTEEN	Our Approach
m1	0.42	0.40	0.66	0.84	0.84	0.86	0.85	0.91	0.89
m2	0.44	0.51	0.84	0.91	0.91	0.91	0.90	0.93	0.92
m3	0.44	0.48	0.79	0.86	0.88	0.89	0.87	0.94	0.92
m4	0.44	0.47	0.72	0.84	0.86	0.86	0.85	0.93	0.91
m5	0.44	0.56	0.75	0.84	0.85	0.87	0.84	0.93	0.90
m6	0.44	0.42	0.73	0.85	0.86	0.86	0.85	0.92	0.90
m7	0.42	0.57	0.81	0.83	0.86	0.82	0.86	0.85	0.90
m8	0.42	0.47	0.78	0.81	0.84	0.83	0.86	0.86	0.91
m9	0.42	0.39	0.77	0.83	0.85	0.86	0.84	0.92	0.91
m10	0.42	0.50	0.73	0.85	0.81	0.83	0.85	0.91	0.89
m11	0.42	0.51	0.77	0.81	0.82	0.82	0.81	0.89	0.90
m12	0.44	0.42	0.76	0.83	0.86	0.86	0.86	0.86	0.93
m13	0.44	0.54	0.69	0.83	0.86	0.86	0.85	0.88	0.94
m14	0.44	0.48	0.77	0.83	0.84	0.87	0.86	0.90	0.93
m15	0.44	0.46	0.75	0.80	0.83	0.85	0.83	0.90	0.92
m16	0.44	0.51	0.72	0.81	0.83	0.82	0.83	0.89	0.92
m17	0.80	0.83	0.93	0.93	0.93	0.93	0.92	0.95	0.95
m18	0.74	0.60	0.88	0.90	0.90	0.92	0.90	0.97	0.93
m19	0.75	0.60	0.86	0.89	0.90	0.91	0.89	0.97	0.92
m20	0.78	0.58	0.87	0.86	0.87	0.89	0.89	0.96	0.91
m21	0.73	0.59	0.84	0.87	0.88	0.90	0.89	0.97	0.90
m22	0.85	0.79	0.89	0.94	0.94	0.94	0.93	0.95	0.96
m23	0.44	0.69	0.87	0.89	0.91	0.91	0.91	0.97	0.94
m24	0.44	0.58	0.86	0.89	0.88	0.90	0.89	0.95	0.94
m25	0.44	0.58	0.85	0.87	0.88	0.89	0.89	0.95	0.93
m26	0.44	0.59	0.85	0.86	0.88	0.90	0.89	0.96	0.92
m27	0.48	0.64	0.87	0.87	0.87	0.88	0.87	0.89	0.91
m28	0.48	0.83	0.60	0.67	0.67	0.65	0.86	0.74	0.82
m29	0.73	0.74	0.70	0.72	0.73	0.74	0.74	0.88	0.82
m30	0.51	0.19	0.13	0.21	0.21	0.21	0.21	0.27	0.92
**Mean ± SD**	0.52 ± 0.14	0.55 ± 0.14	0.77 ± 0.14	0.82 ± 0.13	0.84 ± 0.13	0.84 ± 0.13	0.84 ± 0.13	0.89 ± 0.13	**0.91 ± 0.03**

Table A2. G-Mean Results for SVM Classifier Across 30 Imbalanced Datasets

Dataset	WITHOUT	NEARMISS	RUS	ROS	SMOTE	SMOTETomek	BorderlineSMOTE	SMOTEEN	Our Approach
m1	0.75	0.38	0.59	0.75	0.76	0.77	0.78	0.85	0.84
m2	0.89	0.49	0.71	0.86	0.86	0.87	0.86	0.88	0.89
m3	0.88	0.46	0.55	0.81	0.81	0.83	0.82	0.87	0.88
m4	0.88	0.46	0.53	0.77	0.78	0.81	0.81	0.87	0.87
m5	0.89	0.55	0.67	0.76	0.77	0.81	0.80	0.87	0.88
m6	0.88	0.44	0.60	0.75	0.79	0.79	0.79	0.86	0.88
m7	0.86	0.55	0.73	0.78	0.78	0.78	0.80	0.81	0.87
m8	0.87	0.46	0.75	0.77	0.77	0.79	0.78	0.84	0.88
m9	0.84	0.30	0.73	0.77	0.77	0.79	0.76	0.87	0.89
m10	0.86	0.50	0.65	0.75	0.76	0.80	0.76	0.87	0.86
m11	0.86	0.49	0.72	0.76	0.77	0.80	0.76	0.84	0.88
m12	0.89	0.35	0.73	0.78	0.78	0.79	0.78	0.79	0.89
m13	0.89	0.47	0.79	0.76	0.78	0.80	0.79	0.85	0.91
m14	0.89	0.41	0.71	0.76	0.78	0.80	0.79	0.87	0.91
m15	0.89	0.46	0.66	0.79	0.75	0.77	0.77	0.89	0.90
m16	0.89	0.49	0.70	0.74	0.76	0.79	0.75	0.84	0.90
m17	0.88	0.77	0.84	0.91	0.91	0.91	0.89	0.93	0.94
m18	0.87	0.54	0.83	0.87	0.87	0.90	0.87	0.96	0.91
m19	0.87	0.58	0.80	0.85	0.86	0.88	0.85	0.96	0.91
m20	0.86	0.60	0.77	0.82	0.83	0.86	0.83	0.95	0.89
m21	0.85	0.58	0.87	0.81	0.84	0.87	0.84	0.97	0.87
m22	0.93	0.78	0.88	0.92	0.92	0.93	0.89	0.94	0.95
m23	0.91	0.66	0.83	0.86	0.86	0.89	0.87	0.95	0.93
m24	0.91	0.59	0.82	0.84	0.85	0.86	0.85	0.94	0.92
m25	0.91	0.59	0.80	0.83	0.83	0.86	0.83	0.94	0.92
m26	0.91	0.58	0.83	0.82	0.83	0.86	0.83	0.95	0.91
m27	0.97	0.70	0.81	0.82	0.83	0.84	0.84	0.90	0.90
m28	0.97	0.80	0.22	0.69	0.66	0.67	0.82	0.54	0.82
m29	0.83	0.75	0.67	0.70	0.69	0.70	0.65	0.82	0.77
m30	0.95	0.20	0.12	0.29	0.29	0.29	0.29	0.00	0.90
**Mean $\pm$ Std**	0.89 ± 0.03	0.54 ± 0.14	0.70 ± 0.17	0.78 ± 0.11	0.79 ± 0.11	0.81 ± 0.11	0.79 ± 0.11	0.85 ± 0.18	$\mathbf{0.90 \pm 0.03}$

Table A3. F1-Score Results for SVM Classifier Across 30 Imbalanced Datasets

Dataset	WITHOUT	NEARMISS	RUS	ROS	SMOTE	SMOTETomek	BorderlineSMOTE	SMOTEEN	Our Approach
m1	0.91	0.39	0.62	0.77	0.76	0.79	0.79	0.91	0.89
m2	0.93	0.49	0.75	0.87	0.88	0.88	0.86	0.90	0.92
m3	0.93	0.47	0.58	0.81	0.83	0.84	0.81	0.92	0.92
m4	0.93	0.48	0.54	0.78	0.79	0.82	0.80	0.90	0.91
m5	0.93	0.58	0.71	0.78	0.79	0.83	0.81	0.91	0.90
m6	0.93	0.46	0.62	0.77	0.80	0.81	0.79	0.90	0.90
m7	0.91	0.58	0.75	0.79	0.79	0.80	0.82	0.82	0.91
m8	0.91	0.48	0.76	0.79	0.78	0.80	0.78	0.85	0.91
m9	0.91	0.31	0.75	0.78	0.79	0.82	0.78	0.89	0.91
m10	0.91	0.54	0.68	0.77	0.78	0.82	0.77	0.91	0.89
m11	0.91	0.53	0.74	0.77	0.78	0.81	0.77	0.87	0.90
m12	0.93	0.37	0.75	0.79	0.80	0.80	0.79	0.81	0.93
m13	0.93	0.50	0.81	0.78	0.80	0.81	0.80	0.86	0.93
m14	0.93	0.42	0.74	0.78	0.80	0.81	0.80	0.89	0.93
m15	0.93	0.47	0.69	0.80	0.77	0.79	0.78	0.90	0.92
m16	0.93	0.51	0.71	0.76	0.77	0.80	0.76	0.86	0.93
m17	0.93	0.79	0.85	0.91	0.92	0.92	0.90	0.93	0.95
m18	0.92	0.55	0.84	0.88	0.89	0.92	0.88	0.96	0.93
m19	0.92	0.59	0.81	0.87	0.88	0.90	0.86	0.96	0.92
m20	0.92	0.61	0.78	0.83	0.85	0.88	0.84	0.95	0.91
m21	0.91	0.60	0.88	0.83	0.86	0.89	0.86	0.97	0.90
m22	0.95	0.80	0.89	0.93	0.93	0.93	0.90	0.95	0.96
m23	0.93	0.68	0.85	0.87	0.88	0.91	0.88	0.96	0.94
m24	0.93	0.60	0.84	0.85	0.87	0.88	0.86	0.95	0.93
m25	0.93	0.60	0.81	0.84	0.85	0.87	0.84	0.95	0.93
m26	0.93	0.59	0.85	0.84	0.84	0.87	0.84	0.96	0.92
m27	0.98	0.71	0.83	0.84	0.85	0.85	0.85	0.91	0.92
m28	0.98	0.81	0.23	0.70	0.68	0.69	0.84	0.55	0.83
m29	0.85	0.76	0.68	0.72	0.71	0.71	0.67	0.83	0.78
m30	0.96	0.21	0.13	0.30	0.30	0.30	0.30	0.00	0.91
**Mean ± Std**	0.93 ± 0.02	0.55 ± 0.14	0.72 ± 0.17	0.79 ± 0.11	0.80 ± 0.11	0.82 ± 0.11	0.80 ± 0.11	0.86 ± 0.18	**0.91 ± 0.03**

Table A4. Area Under the Curve (AUC) Scores for SVM Classifier Across Resampling Methods

Dataset	WITHOUT	NEARMISS	RUS	ROS	SMOTE	SMOTETomek	BorderlineSMOTE	SMOTEEN	Our Approach
m1	0.80	0.49	0.73	0.86	0.87	0.89	0.86	0.97	0.95
m2	0.93	0.58	0.88	0.93	0.94	0.94	0.94	0.96	0.98
m3	0.78	0.54	0.78	0.91	0.91	0.92	0.90	0.98	0.97
m4	0.73	0.55	0.80	0.88	0.89	0.91	0.88	0.96	0.97
m5	0.68	0.58	0.83	0.87	0.88	0.89	0.87	0.95	0.96
m6	0.65	0.43	0.74	0.85	0.87	0.88	0.86	0.95	0.96
m7	0.83	0.54	0.85	0.89	0.90	0.90	0.88	0.92	0.96
m8	0.83	0.48	0.86	0.88	0.89	0.90	0.87	0.94	0.97
m9	0.82	0.39	0.80	0.87	0.87	0.90	0.86	0.96	0.96
m10	0.85	0.47	0.86	0.87	0.88	0.90	0.87	0.97	0.96
m11	0.80	0.51	0.83	0.87	0.87	0.89	0.86	0.96	0.96
m12	0.92	0.42	0.84	0.91	0.91	0.91	0.89	0.92	0.97
m13	0.91	0.51	0.84	0.88	0.89	0.91	0.87	0.94	0.97
m14	0.89	0.45	0.83	0.86	0.88	0.89	0.87	0.96	0.97
m15	0.86	0.44	0.83	0.86	0.87	0.89	0.87	0.96	0.97
m16	0.86	0.51	0.79	0.86	0.87	0.89	0.86	0.95	0.96
m17	0.95	0.89	0.93	0.97	0.97	0.97	0.96	0.98	0.99
m18	0.93	0.72	0.94	0.94	0.94	0.95	0.93	0.99	0.97
m19	0.92	0.65	0.92	0.94	0.94	0.95	0.92	0.99	0.97
m20	0.90	0.64	0.89	0.92	0.92	0.94	0.91	0.98	0.97
m21	0.91	0.64	0.88	0.92	0.92	0.93	0.91	0.99	0.97
m22	0.94	0.88	0.95	0.98	0.98	0.98	0.96	0.98	0.99
m23	0.92	0.71	0.93	0.94	0.95	0.96	0.93	0.98	0.98
m24	0.90	0.63	0.92	0.93	0.94	0.95	0.92	0.99	0.97
m25	0.88	0.59	0.91	0.92	0.93	0.93	0.91	0.97	0.97
m26	0.88	0.60	0.88	0.91	0.92	0.93	0.90	0.98	0.97
m27	0.74	0.71	0.86	0.94	0.95	0.94	0.95	0.99	0.98
m28	0.52	0.77	0.66	0.70	0.72	0.73	0.90	0.83	0.90
m29	0.80	0.79	0.79	0.81	0.82	0.83	0.78	0.94	0.61
m30	0.00	0.00	0.00	0.67	0.63	0.62	0.67	0.60	0.95
**Mean $\pm$ Std**	0.81 $\pm$ 0.18	0.57 $\pm$ 0.17	0.82 $\pm$ 0.17	0.88 $\pm$ 0.07	0.89 $\pm$ 0.07	0.90 $\pm$ 0.07	0.89 $\pm$ 0.06	0.95 $\pm$ 0.07	**0.95 $\pm$ 0.07**

Table A5. Recall scores using SVM classifier across 30 datasets and 9 resampling techniques

Dataset	WITHOUT	NEARMISS	RUS	ROS	SMOTE	SMOTETomek	BorderlineSMOTE	SMOTEEN	Our Approach
m1	0.50	0.42	0.65	0.82	0.82	0.83	0.82	0.91	0.89
m2	0.50	0.51	0.78	0.89	0.89	0.89	0.88	0.91	0.92
m3	0.50	0.48	0.74	0.83	0.84	0.87	0.84	0.91	0.92
m4	0.50	0.48	0.68	0.81	0.83	0.84	0.82	0.91	0.91
m5	0.50	0.54	0.69	0.82	0.82	0.85	0.82	0.91	0.90
m6	0.50	0.43	0.69	0.82	0.82	0.84	0.83	0.91	0.91
m7	0.50	0.57	0.75	0.81	0.83	0.80	0.83	0.84	0.90
m8	0.50	0.47	0.76	0.80	0.81	0.83	0.82	0.85	0.91
m9	0.50	0.41	0.76	0.79	0.82	0.83	0.82	0.91	0.91
m10	0.50	0.50	0.76	0.81	0.82	0.82	0.81	0.90	0.90
m11	0.50	0.50	0.72	0.79	0.80	0.81	0.80	0.87	0.90
m12	0.50	0.42	0.77	0.82	0.83	0.83	0.82	0.85	0.93
m13	0.50	0.53	0.73	0.82	0.83	0.83	0.82	0.87	0.93
m14	0.50	0.47	0.79	0.81	0.82	0.84	0.82	0.91	0.93
m15	0.50	0.46	0.73	0.81	0.80	0.82	0.80	0.90	0.91
m16	0.50	0.51	0.72	0.80	0.81	0.81	0.80	0.89	0.93
m17	0.76	0.81	0.89	0.92	0.92	0.92	0.91	0.94	0.95
m18	0.70	0.59	0.90	0.89	0.91	0.92	0.89	0.97	0.93
m19	0.71	0.60	0.83	0.88	0.89	0.90	0.87	0.96	0.93
m20	0.66	0.56	0.84	0.86	0.87	0.88	0.86	0.96	0.90
m21	0.65	0.59	0.88	0.86	0.88	0.90	0.87	0.97	0.90
m22	0.66	0.77	0.90	0.93	0.93	0.93	0.91	0.95	0.96
m23	0.50	0.68	0.89	0.89	0.89	0.91	0.89	0.96	0.94
m24	0.50	0.58	0.85	0.87	0.89	0.90	0.88	0.96	0.94
m25	0.50	0.57	0.81	0.86	0.86	0.88	0.86	0.95	0.93
m26	0.50	0.58	0.86	0.86	0.87	0.88	0.87	0.96	0.92
m27	0.50	0.62	0.89	0.86	0.87	0.87	0.86	0.92	0.91
m28	0.50	0.77	0.59	0.65	0.66	0.67	0.84	0.68	0.81
m29	0.62	0.72	0.70	0.72	0.73	0.74	0.71	0.86	0.73
m30	0.55	0.42	0.40	0.50	0.50	0.50	0.50	0.50	0.92
**Avg $\pm$ Std**	0.54 $\pm$ 0.08	0.55 $\pm$ 0.11	0.76 $\pm$ 0.11	0.82 $\pm$ 0.08	0.83 $\pm$ 0.08	0.84 $\pm$ 0.08	0.83 $\pm$ 0.07	0.89 $\pm$ 0.09	**0.91 $\pm$ 0.04**

Table A6. Recall results using Naive Bayes across different sampling techniques

Dataset	WITHOUT	NEARMISS	RUS	ROS	SMOTE	SMOTETomek	BorderlineSMOTE	SMOTEEN	Our Approach
m1	0.50	0.44	0.72	0.73	0.75	0.78	0.77	0.88	0.87
m2	0.50	0.63	0.85	0.87	0.87	0.88	0.88	0.89	0.92
m3	0.50	0.57	0.79	0.82	0.83	0.85	0.84	0.91	0.92
m4	0.50	0.57	0.75	0.75	0.78	0.82	0.82	0.85	0.90
m5	0.50	0.54	0.73	0.75	0.76	0.77	0.79	0.87	0.89
m6	0.50	0.47	0.75	0.75	0.76	0.78	0.77	0.85	0.89
m7	0.50	0.52	0.75	0.76	0.78	0.78	0.75	0.80	0.89
m8	0.50	0.53	0.73	0.75	0.75	0.78	0.75	0.83	0.90
m9	0.50	0.47	0.74	0.75	0.77	0.77	0.76	0.84	0.91
m10	0.50	0.46	0.77	0.76	0.77	0.77	0.74	0.88	0.89
m11	0.50	0.56	0.78	0.74	0.76	0.78	0.76	0.83	0.90
m12	0.50	0.43	0.75	0.75	0.77	0.75	0.74	0.78	0.92
m13	0.50	0.56	0.74	0.75	0.76	0.78	0.75	0.82	0.93
m14	0.50	0.49	0.76	0.75	0.76	0.77	0.75	0.86	0.93
m15	0.50	0.48	0.76	0.75	0.74	0.77	0.75	0.85	0.91
m16	0.50	0.56	0.78	0.73	0.75	0.75	0.75	0.83	0.92
m17	0.51	0.60	0.86	0.88	0.87	0.88	0.85	0.91	0.90
m18	0.50	0.54	0.84	0.85	0.88	0.89	0.85	0.93	0.90
m19	0.50	0.59	0.84	0.84	0.85	0.85	0.83	0.92	0.90
m20	0.50	0.50	0.77	0.81	0.82	0.83	0.79	0.91	0.89
m21	0.50	0.61	0.83	0.83	0.85	0.84	0.82	0.91	0.90
m22	0.50	0.50	0.84	0.89	0.89	0.89	0.79	0.91	0.92
m23	0.50	0.61	0.84	0.85	0.87	0.87	0.84	0.93	0.92
m24	0.50	0.58	0.81	0.84	0.84	0.86	0.83	0.91	0.92
m25	0.50	0.56	0.80	0.82	0.83	0.84	0.80	0.90	0.91
m26	0.50	0.57	0.78	0.81	0.82	0.82	0.82	0.91	0.92
m27	0.83	0.68	0.67	0.81	0.66	0.68	0.66	0.91	0.91
m28	0.54	0.82	0.64	0.71	0.71	0.72	0.84	0.75	0.81
m29	0.52	0.57	0.51	0.51	0.52	0.52	0.52	0.54	0.73
m30	0.57	0.61	0.58	0.70	0.69	0.67	0.69	0.72	0.88
**Mean $\pm$ Std**	0.52 ± 0.06	0.55 ± 0.08	0.76 ± 0.08	0.78 ± 0.07	0.78 ± 0.08	0.79 ± 0.08	0.78 ± 0.07	0.85 ± 0.08	$\mathbf{0.90 \pm 0.04}$

Table A7. Precision results using Naive Bayes across different sampling techniques

Dataset	WITHOUT	NEARMISS	RUS	ROS	SMOTE	SMOTETomek	BorderlineSMOTE	SMOTEEN	Our Approach
m1	0.42	0.43	0.71	0.73	0.77	0.79	0.78	0.85	0.87
m2	0.44	0.64	0.86	0.88	0.88	0.89	0.89	0.91	0.91
m3	0.44	0.58	0.78	0.82	0.83	0.85	0.83	0.91	0.92
m4	0.44	0.58	0.74	0.75	0.79	0.80	0.82	0.84	0.90
m5	0.44	0.54	0.76	0.76	0.75	0.77	0.78	0.87	0.89
m6	0.44	0.46	0.70	0.75	0.76	0.78	0.77	0.86	0.89
m7	0.42	0.52	0.75	0.78	0.78	0.79	0.74	0.76	0.89
m8	0.42	0.53	0.74	0.75	0.76	0.78	0.73	0.84	0.90
m9	0.42	0.46	0.74	0.75	0.75	0.76	0.75	0.87	0.91
m10	0.42	0.45	0.80	0.76	0.76	0.78	0.75	0.86	0.90
m11	0.42	0.56	0.75	0.75	0.76	0.77	0.75	0.84	0.90
m12	0.44	0.42	0.73	0.76	0.76	0.76	0.76	0.81	0.92
m13	0.44	0.56	0.78	0.77	0.77	0.76	0.76	0.79	0.93
m14	0.44	0.50	0.76	0.75	0.76	0.77	0.76	0.84	0.93
m15	0.44	0.48	0.75	0.76	0.76	0.77	0.74	0.85	0.91
m16	0.44	0.56	0.70	0.73	0.74	0.76	0.74	0.82	0.92
m17	0.47	0.61	0.85	0.88	0.89	0.88	0.84	0.90	0.90
m18	0.42	0.54	0.84	0.87	0.88	0.89	0.82	0.93	0.90
m19	0.42	0.59	0.85	0.84	0.85	0.87	0.83	0.92	0.90
m20	0.42	0.50	0.79	0.81	0.82	0.84	0.78	0.90	0.89
m21	0.42	0.61	0.80	0.83	0.82	0.85	0.82	0.92	0.89
m22	0.44	0.50	0.88	0.88	0.89	0.89	0.81	0.91	0.92
m23	0.44	0.61	0.88	0.85	0.86	0.88	0.81	0.93	0.93
m24	0.44	0.58	0.85	0.83	0.85	0.86	0.83	0.90	0.92
m25	0.44	0.56	0.82	0.82	0.82	0.84	0.81	0.90	0.92
m26	0.44	0.57	0.83	0.80	0.82	0.82	0.82	0.90	0.91
m27	0.58	0.76	0.76	0.83	0.79	0.78	0.77	0.89	0.92
m28	0.54	0.82	0.59	0.70	0.72	0.73	0.85	0.77	0.83
m29	0.61	0.75	0.66	0.69	0.71	0.70	0.72	0.78	0.83
m30	0.52	0.56	0.28	0.76	0.71	0.72	0.78	0.75	0.89
**Mean ± Std**	0.45 ± 0.05	0.56 ± 0.09	0.76 ± 0.11	0.79 ± 0.05	0.79 ± 0.05	0.80 ± 0.05	0.79 ± 0.04	0.86 ± 0.05	**0.90 ± 0.02**

Table A8. F1-score results using Naive Bayes across different sampling techniques

Dataset	WITHOUT	NEARMISS	RUS	ROS	SMOTE	SMOTETomek	BorderlineSMOTE	SMOTEEN	Our Approach
m1	0.91	0.40	0.75	0.72	0.74	0.81	0.74	0.86	0.88
m2	0.93	0.61	0.84	0.87	0.88	0.87	0.88	0.89	0.92
m3	0.93	0.57	0.79	0.80	0.84	0.85	0.83	0.90	0.92
m4	0.93	0.58	0.75	0.75	0.78	0.78	0.80	0.84	0.90
m5	0.93	0.55	0.70	0.75	0.76	0.78	0.77	0.87	0.89
m6	0.93	0.41	0.70	0.73	0.75	0.77	0.77	0.84	0.89
m7	0.91	0.49	0.77	0.77	0.78	0.74	0.75	0.79	0.90
m8	0.91	0.49	0.74	0.74	0.75	0.77	0.74	0.82	0.90
m9	0.91	0.38	0.78	0.75	0.76	0.78	0.76	0.86	0.91
m10	0.91	0.44	0.72	0.76	0.76	0.76	0.74	0.88	0.90
m11	0.91	0.53	0.72	0.73	0.75	0.77	0.74	0.83	0.89
m12	0.93	0.37	0.76	0.77	0.78	0.77	0.76	0.77	0.92
m13	0.93	0.50	0.73	0.75	0.77	0.77	0.74	0.80	0.93
m14	0.93	0.42	0.76	0.76	0.76	0.78	0.76	0.85	0.93
m15	0.93	0.48	0.73	0.75	0.76	0.77	0.74	0.84	0.91
m16	0.93	0.56	0.76	0.74	0.74	0.77	0.75	0.82	0.92
m17	0.91	0.59	0.89	0.87	0.88	0.87	0.85	0.90	0.90
m18	0.91	0.53	0.88	0.86	0.86	0.88	0.82	0.94	0.90
m19	0.91	0.59	0.82	0.83	0.84	0.86	0.83	0.93	0.90
m20	0.91	0.42	0.79	0.81	0.83	0.84	0.77	0.91	0.89
m21	0.91	0.62	0.78	0.83	0.83	0.85	0.81	0.90	0.90
m22	0.93	0.47	0.89	0.87	0.89	0.89	0.81	0.89	0.92
m23	0.93	0.61	0.84	0.85	0.86	0.88	0.82	0.93	0.93
m24	0.93	0.59	0.84	0.83	0.84	0.85	0.82	0.91	0.92
m25	0.93	0.52	0.80	0.81	0.82	0.83	0.79	0.90	0.92
m26	0.93	0.56	0.80	0.80	0.82	0.83	0.81	0.90	0.92
m27	0.86	0.72	0.73	0.76	0.48	0.53	0.49	0.92	0.92
m28	0.96	0.84	0.63	0.73	0.70	0.70	0.81	0.70	0.83
m29	0.09	0.70	0.04	0.05	0.11	0.10	0.09	0.30	0.79
m30	0.58	0.67	0.63	0.57	0.57	0.60	0.58	0.65	0.90
**Mean $\pm$ Std**	0.88 $\pm$ 0.16	0.54 $\pm$ 0.11	0.75 $\pm$ 0.15	0.75 $\pm$ 0.15	0.76 $\pm$ 0.15	0.77 $\pm$ 0.15	0.75 $\pm$ 0.14	0.84 $\pm$ 0.12	**0.90 $\pm$ 0.03**

Table A9. AUC performance of Naive Bayes classifier across different resampling techniques

Dataset	WITHOUT	NEARMISS	RUS	ROS	SMOTE	SMOTETomek	BorderlineSMOTE	SMOTEEN	Our Approach
m1	0.84	0.47	0.82	0.83	0.86	0.87	0.86	0.95	0.95
m2	0.92	0.72	0.91	0.92	0.93	0.93	0.92	0.94	0.97
m3	0.88	0.62	0.85	0.89	0.90	0.91	0.90	0.96	0.97
m4	0.84	0.58	0.85	0.85	0.87	0.89	0.88	0.95	0.96
m5	0.84	0.55	0.82	0.85	0.86	0.88	0.87	0.94	0.95
m6	0.82	0.43	0.78	0.82	0.84	0.86	0.85	0.93	0.95
m7	0.85	0.53	0.84	0.85	0.86	0.85	0.85	0.89	0.96
m8	0.84	0.52	0.87	0.85	0.85	0.87	0.81	0.92	0.96
m9	0.84	0.42	0.83	0.86	0.86	0.87	0.85	0.96	0.97
m10	0.84	0.43	0.84	0.84	0.86	0.87	0.85	0.94	0.96
m11	0.83	0.56	0.83	0.84	0.84	0.88	0.84	0.95	0.96
m12	0.85	0.41	0.86	0.86	0.87	0.86	0.85	0.89	0.97
m13	0.85	0.56	0.89	0.86	0.86	0.88	0.83	0.91	0.97
m14	0.85	0.50	0.85	0.85	0.87	0.87	0.86	0.95	0.98
m15	0.84	0.45	0.84	0.85	0.86	0.87	0.85	0.94	0.97
m16	0.82	0.53	0.83	0.84	0.84	0.86	0.84	0.93	0.97
m17	0.92	0.69	0.98	0.93	0.93	0.94	0.91	0.95	0.97
m18	0.91	0.58	0.89	0.91	0.92	0.93	0.88	0.96	0.96
m19	0.91	0.60	0.90	0.90	0.92	0.92	0.89	0.98	0.97
m20	0.89	0.55	0.88	0.89	0.90	0.91	0.86	0.97	0.96
m21	0.89	0.62	0.90	0.90	0.91	0.92	0.88	0.98	0.97
m22	0.93	0.57	0.91	0.93	0.93	0.93	0.90	0.95	0.98
m23	0.92	0.64	0.89	0.91	0.92	0.93	0.89	0.97	0.98
m24	0.90	0.60	0.89	0.91	0.91	0.92	0.89	0.97	0.97
m25	0.89	0.58	0.89	0.89	0.89	0.90	0.87	0.96	0.97
m26	0.89	0.59	0.86	0.89	0.89	0.91	0.88	0.97	0.97
m27	0.88	0.82	0.85	0.91	0.91	0.91	0.90	0.95	0.97
m28	0.66	0.87	0.59	0.77	0.79	0.80	0.92	0.83	0.90
m29	0.78	0.77	0.78	0.77	0.78	0.79	0.75	0.93	0.72
m30	0.73	0.60	0.78	0.78	0.86	0.85	0.85	0.88	0.93
**Mean $\pm$ Std**	**0.86 $\pm$ 0.06**	**0.58 $\pm$ 0.11**	**0.85 $\pm$ 0.07**	**0.86 $\pm$ 0.04**	**0.88 $\pm$ 0.04**	**0.89 $\pm$ 0.04**	**0.87 $\pm$ 0.03**	**0.94 $\pm$ 0.03**	**0.95 $\pm$ 0.05**

Table A10. G-Mean performance of Naive Bayes across different resampling techniques

Dataset	WITHOUT	NEARMISS	RUS	ROS	SMOTE	SMOTETomek	BorderlineSMOTE	SMOTEEN	Our Approach
m1	0.50	0.44	0.71	0.75	0.76	0.77	0.77	0.83	0.88
m2	0.50	0.63	0.85	0.88	0.89	0.88	0.88	0.89	0.92
m3	0.50	0.57	0.79	0.82	0.83	0.85	0.84	0.90	0.92
m4	0.50	0.57	0.76	0.74	0.79	0.81	0.80	0.85	0.90
m5	0.50	0.54	0.76	0.74	0.76	0.78	0.78	0.86	0.89
m6	0.50	0.47	0.78	0.76	0.77	0.76	0.79	0.83	0.89
m7	0.50	0.52	0.75	0.76	0.76	0.76	0.74	0.79	0.90
m8	0.50	0.53	0.79	0.75	0.78	0.77	0.76	0.82	0.90
m9	0.50	0.47	0.71	0.75	0.76	0.77	0.77	0.87	0.91
m10	0.50	0.46	0.75	0.74	0.74	0.77	0.75	0.90	0.90
m11	0.50	0.56	0.76	0.75	0.75	0.77	0.73	0.83	0.89
m12	0.50	0.43	0.75	0.78	0.78	0.76	0.75	0.78	0.92
m13	0.50	0.56	0.79	0.75	0.77	0.78	0.74	0.83	0.93
m14	0.50	0.49	0.72	0.75	0.76	0.77	0.76	0.85	0.93
m15	0.50	0.48	0.73	0.73	0.75	0.77	0.73	0.84	0.91
m16	0.50	0.56	0.72	0.73	0.75	0.75	0.73	0.83	0.92
m17	0.51	0.60	0.88	0.87	0.88	0.88	0.85	0.91	0.90
m18	0.50	0.54	0.84	0.86	0.88	0.87	0.83	0.94	0.90
m19	0.50	0.59	0.85	0.84	0.84	0.85	0.84	0.92	0.90
m20	0.50	0.50	0.80	0.81	0.83	0.84	0.80	0.89	0.89
m21	0.50	0.61	0.81	0.83	0.83	0.85	0.82	0.91	0.90
m22	0.50	0.50	0.87	0.88	0.89	0.88	0.79	0.90	0.92
m23	0.50	0.61	0.84	0.86	0.87	0.87	0.83	0.94	0.93
m24	0.50	0.58	0.76	0.82	0.84	0.86	0.83	0.91	0.92
m25	0.50	0.56	0.82	0.82	0.83	0.83	0.79	0.91	0.92
m26	0.50	0.57	0.82	0.81	0.82	0.83	0.83	0.89	0.92
m27	0.83	0.68	0.74	0.80	0.58	0.69	0.64	0.87	0.92
m28	0.54	0.82	0.60	0.70	0.72	0.72	0.83	0.74	0.83
m29	0.52	0.57	0.51	0.51	0.53	0.53	0.52	0.53	0.79
m30	0.57	0.61	0.59	0.69	0.68	0.68	0.70	0.72	0.90
**Mean ± Std**	**0.52 ± 0.06**	**0.55 ± 0.08**	**0.76 ± 0.08**	**0.78 ± 0.07**	**0.78 ± 0.08**	**0.79 ± 0.07**	**0.77 ± 0.07**	**0.85 ± 0.08**	**0.90 ± 0.03**

Table A11. AUC performance of Random Forest classifier across various sampling techniques.

Dataset	WITHOUT	NEARMISS	RUS	ROS	SMOTE	SMOTETomek	Bord.SMOTE	SMOTEEN	Proposed
m1	0.66	0.56	0.76	0.93	0.86	0.89	0.85	0.97	0.97
m2	0.99	0.91	0.96	1.00	0.99	0.99	1.00	0.98	1.00
m3	0.77	0.71	0.85	0.97	0.89	0.94	0.89	0.97	0.98
m4	0.70	0.63	0.75	0.95	0.87	0.91	0.87	0.97	0.97
m5	0.71	0.60	0.77	0.95	0.87	0.91	0.87	0.97	0.97
m6	0.65	0.49	0.69	0.95	0.87	0.90	0.88	0.97	0.97
m7	0.85	0.77	0.88	0.97	0.95	0.96	0.96	0.98	0.99
m8	0.78	0.63	0.79	0.96	0.91	0.92	0.91	0.97	0.98
m9	0.69	0.54	0.78	0.95	0.88	0.92	0.87	0.97	0.97
m10	0.69	0.54	0.77	0.95	0.86	0.89	0.86	0.98	0.98
m11	0.63	0.45	0.68	0.93	0.85	0.90	0.86	0.96	0.97
m12	0.83	0.71	0.83	0.98	0.96	0.96	0.97	0.97	0.99
m13	0.75	0.67	0.83	0.96	0.93	0.94	0.92	0.98	0.99
m14	0.64	0.57	0.72	0.96	0.91	0.92	0.89	0.97	0.98
m15	0.62	0.51	0.76	0.96	0.85	0.91	0.87	0.97	0.98
m16	0.64	0.50	0.77	0.96	0.87	0.91	0.87	0.97	0.98
m17	0.93	0.86	0.92	0.99	0.99	0.98	0.99	0.99	1.00
m18	0.79	0.68	0.85	0.97	0.91	0.94	0.91	0.98	0.98
m19	0.78	0.66	0.80	0.96	0.91	0.92	0.90	0.97	0.98
m20	0.72	0.50	0.72	0.94	0.87	0.92	0.89	0.98	0.96
m21	0.72	0.54	0.78	0.94	0.89	0.92	0.89	0.99	0.97
m22	0.91	0.83	0.95	0.99	0.99	0.99	0.98	0.99	0.99
m23	0.80	0.71	0.77	0.98	0.92	0.94	0.92	0.98	0.98
m24	0.69	0.64	0.78	0.97	0.91	0.93	0.90	0.98	0.97
m25	0.72	0.53	0.77	0.96	0.89	0.92	0.89	0.98	0.97
m26	0.70	0.50	0.75	0.96	0.90	0.91	0.88	0.98	0.97
m27	0.68	0.53	0.72	0.97	0.96	0.97	0.96	1.00	0.99
m28	0.58	0.76	0.67	0.98	0.92	0.93	0.96	0.93	0.93
m29	0.67	0.63	0.66	0.85	0.77	0.78	0.78	0.90	0.89
m30	0.57	0.48	0.74	0.98	0.95	0.96	0.94	0.96	0.95
**Avg $\pm$ Std**	0.73±0.10	0.62±0.12	0.78±0.08	0.96±0.03	0.90±0.05	0.93±0.04	0.90±0.05	0.97±0.02	0.97±0.02

Table A12. Recall values for Random Forest classifier across different sampling methods

Dataset	WITHOUT	NEARMISS	RUS	ROS	SMOTE	SMOTETomek	BorderlineSMOTE	SMOTEEN	Our Approach
m1	0.68	0.55	0.76	0.94	0.86	0.88	0.85	0.97	0.90
m2	0.99	0.91	0.93	1.00	0.99	0.99	0.99	0.99	0.98
m3	0.77	0.70	0.78	0.97	0.91	0.92	0.91	0.97	0.94
m4	0.70	0.61	0.74	0.95	0.89	0.90	0.87	0.97	0.93
m5	0.71	0.59	0.77	0.96	0.87	0.91	0.87	0.97	0.92
m6	0.62	0.49	0.77	0.95	0.87	0.91	0.86	0.97	0.92
m7	0.84	0.77	0.86	0.97	0.96	0.96	0.95	0.98	0.96
m8	0.78	0.62	0.80	0.95	0.91	0.91	0.91	0.98	0.93
m9	0.69	0.52	0.77	0.95	0.88	0.91	0.88	0.97	0.92
m10	0.70	0.53	0.76	0.93	0.86	0.88	0.85	0.98	0.91
m11	0.64	0.45	0.75	0.93	0.85	0.91	0.88	0.97	0.91
m12	0.83	0.70	0.84	0.98	0.97	0.96	0.96	0.98	0.96
m13	0.76	0.67	0.83	0.97	0.92	0.93	0.92	0.97	0.94
m14	0.63	0.56	0.81	0.97	0.90	0.91	0.89	0.97	0.93
m15	0.64	0.51	0.74	0.96	0.86	0.91	0.86	0.98	0.94
m16	0.63	0.51	0.75	0.95	0.86	0.91	0.87	0.97	0.93
m17	0.93	0.87	0.92	0.99	0.98	0.98	0.98	0.99	0.97
m18	0.79	0.69	0.78	0.97	0.91	0.93	0.93	0.98	0.93
m19	0.78	0.65	0.82	0.95	0.91	0.90	0.90	0.98	0.93
m20	0.73	0.53	0.79	0.95	0.88	0.94	0.89	0.97	0.90
m21	0.72	0.56	0.84	0.95	0.88	0.91	0.88	0.97	0.92
m22	0.93	0.83	0.90	0.99	0.99	0.99	0.99	0.99	0.98
m23	0.80	0.70	0.80	0.97	0.93	0.95	0.92	0.97	0.95
m24	0.72	0.62	0.74	0.97	0.91	0.92	0.91	0.97	0.93
m25	0.72	0.52	0.85	0.96	0.90	0.92	0.89	0.98	0.92
m26	0.69	0.53	0.78	0.95	0.88	0.91	0.89	0.98	0.94
m27	0.60	0.60	0.76	0.95	0.95	0.94	0.95	0.99	0.97
m28	0.55	0.74	0.62	0.98	0.91	0.92	0.96	0.93	0.85
m29	0.66	0.63	0.66	0.84	0.76	0.79	0.75	0.90	0.82
m30	0.59	0.49	0.75	0.97	0.96	0.94	0.92	0.96	0.89
**Mean ± SD**	0.73 ± 0.10	0.62 ± 0.12	0.79 ± 0.07	0.96 ± 0.03	0.90 ± 0.05	0.92 ± 0.04	0.90 ± 0.05	0.97 ± 0.02	0.93 ± 0.03

Table A13. Precision of Random Forest across sampling methods

Dataset	WITHOUT	NEARMISS	RUS	ROS	SMOTE	SMOTETomek	Bord.SMOTE	SMOTEEN	Ours
m1	0.69	0.57	0.82	0.95	0.86	0.88	0.86	0.98	0.91
m2	0.98	0.91	0.97	1.00	1.00	0.98	1.00	1.00	0.98
m3	0.76	0.70	0.81	0.97	0.90	0.92	0.90	0.97	0.94
m4	0.69	0.62	0.76	0.96	0.88	0.91	0.86	0.97	0.93
m5	0.70	0.61	0.74	0.96	0.87	0.90	0.87	0.97	0.92
m6	0.63	0.49	0.79	0.96	0.86	0.89	0.87	0.97	0.92
m7	0.83	0.78	0.85	0.98	0.95	0.96	0.96	0.97	0.95
m8	0.75	0.61	0.80	0.96	0.91	0.93	0.91	0.97	0.93
m9	0.71	0.53	0.76	0.95	0.88	0.90	0.89	0.98	0.91
m10	0.73	0.54	0.76	0.94	0.89	0.89	0.83	0.98	0.92
m11	0.65	0.47	0.77	0.94	0.85	0.91	0.86	0.98	0.91
m12	0.84	0.71	0.90	0.97	0.97	0.97	0.96	0.98	0.96
m13	0.74	0.68	0.80	0.97	0.93	0.93	0.93	0.97	0.95
m14	0.64	0.55	0.80	0.97	0.88	0.91	0.89	0.97	0.93
m15	0.64	0.49	0.80	0.96	0.88	0.90	0.89	0.98	0.93
m16	0.66	0.51	0.81	0.96	0.87	0.90	0.89	0.97	0.93
m17	0.93	0.88	0.93	0.99	0.99	0.98	0.99	0.99	0.98
m18	0.77	0.70	0.84	0.97	0.93	0.96	0.93	0.98	0.93
m19	0.77	0.67	0.86	0.96	0.91	0.93	0.90	0.98	0.93
m20	0.72	0.51	0.80	0.95	0.90	0.90	0.88	0.97	0.91
m21	0.72	0.55	0.83	0.95	0.87	0.91	0.88	0.97	0.92
m22	0.94	0.83	0.89	1.00	0.99	0.99	0.98	0.99	0.98
m23	0.77	0.71	0.77	0.97	0.93	0.94	0.92	0.98	0.94
m24	0.73	0.61	0.78	0.97	0.90	0.92	0.89	0.98	0.94
m25	0.71	0.52	0.79	0.96	0.88	0.91	0.90	0.96	0.92
m26	0.69	0.49	0.80	0.96	0.88	0.92	0.89	0.98	0.93
m27	0.59	0.59	0.72	0.95	0.94	0.94	0.94	0.99	0.97
m28	0.53	0.71	0.49	0.98	0.92	0.92	0.96	0.94	0.86
m29	0.67	0.63	0.66	0.86	0.77	0.80	0.77	0.90	0.82
m30	0.58	0.55	0.75	0.97	0.94	0.93	0.94	0.97	0.89
**Avg $\pm$ Std**	0.73 ± 0.10	0.62 ± 0.12	0.79 ± 0.08	0.96 ± 0.02	0.90 ± 0.05	0.92 ± 0.04	0.90 ± 0.05	0.97 ± 0.02	0.93 ± 0.03

Table A14. G-Mean performance using Random Forest across different resampling techniques

Dataset	WITHOUT	NEARMISS	RUS	ROS	SMOTE	SMOTETomek	BorderlineSMOTE	SMOTEEN	Our Approach
m1	0.68	0.54	0.75	0.95	0.87	0.88	0.85	0.99	0.91
m2	0.99	0.91	0.91	1.00	0.99	0.99	1.00	0.98	0.98
m3	0.77	0.71	0.81	0.97	0.89	0.91	0.91	0.97	0.94
m4	0.70	0.61	0.78	0.96	0.87	0.89	0.87	0.97	0.93
m5	0.70	0.59	0.80	0.95	0.86	0.91	0.88	0.97	0.92
m6	0.62	0.48	0.80	0.95	0.86	0.90	0.85	0.98	0.92
m7	0.84	0.78	0.82	0.97	0.96	0.96	0.96	0.98	0.95
m8	0.77	0.63	0.81	0.96	0.91	0.93	0.92	0.97	0.93
m9	0.70	0.53	0.80	0.95	0.89	0.91	0.88	0.99	0.91
m10	0.70	0.52	0.78	0.93	0.85	0.89	0.86	0.97	0.92
m11	0.62	0.43	0.72	0.92	0.87	0.89	0.87	0.97	0.91
m12	0.82	0.68	0.87	0.97	0.97	0.96	0.97	0.99	0.96
m13	0.74	0.68	0.83	0.97	0.91	0.93	0.92	0.98	0.95
m14	0.65	0.54	0.76	0.97	0.89	0.91	0.91	0.98	0.93
m15	0.62	0.49	0.77	0.95	0.86	0.91	0.87	0.98	0.93
m16	0.60	0.51	0.75	0.95	0.86	0.90	0.88	0.97	0.93
m17	0.94	0.87	0.89	0.99	0.98	0.98	0.98	0.98	0.98
m18	0.79	0.69	0.80	0.97	0.91	0.95	0.93	0.97	0.93
m19	0.78	0.66	0.83	0.96	0.90	0.91	0.90	0.97	0.93
m20	0.73	0.53	0.82	0.94	0.88	0.91	0.87	0.97	0.91
m21	0.72	0.55	0.71	0.94	0.88	0.91	0.85	0.97	0.92
m22	0.92	0.84	0.92	0.99	0.99	0.99	0.99	0.99	0.98
m23	0.81	0.71	0.78	0.97	0.92	0.95	0.93	0.98	0.94
m24	0.74	0.62	0.79	0.97	0.91	0.93	0.91	0.98	0.94
m25	0.73	0.53	0.81	0.96	0.90	0.92	0.90	0.98	0.92
m26	0.70	0.52	0.82	0.95	0.88	0.91	0.87	0.98	0.93
m27	0.60	0.61	0.71	0.95	0.94	0.93	0.95	1.00	0.97
m28	0.56	0.74	0.65	0.98	0.91	0.91	0.96	0.93	0.86
m29	0.67	0.62	0.65	0.86	0.76	0.79	0.77	0.89	0.82
m30	0.58	0.54	0.81	0.98	0.95	0.94	0.95	0.97	0.89
**Mean ± Std**	0.73 ± 0.11	0.62 ± 0.12	0.79 ± 0.06	0.96 ± 0.03	0.90 ± 0.05	0.92 ± 0.04	0.91 ± 0.05	0.97 ± 0.02	**0.93 ± 0.03**

Table A15. F1-score results using Random Forest across all datasets

Dataset	WITHOUT	NEARMISS	RUS	ROS	SMOTE	SMOTETomek	BorderlineSMOTE	SMOTEEN	Our Approach
m1	0.89	0.54	0.72	0.94	0.85	0.89	0.86	0.97	0.90
m2	1.00	0.90	0.93	1.00	0.99	0.99	1.00	0.98	0.98
m3	0.94	0.71	0.74	0.97	0.89	0.92	0.90	0.97	0.94
m4	0.92	0.64	0.79	0.96	0.88	0.89	0.87	0.96	0.93
m5	0.93	0.61	0.74	0.95	0.87	0.91	0.87	0.97	0.92
m6	0.91	0.48	0.78	0.95	0.86	0.89	0.88	0.96	0.92
m7	0.95	0.76	0.88	0.98	0.96	0.95	0.96	0.96	0.96
m8	0.92	0.59	0.78	0.96	0.91	0.93	0.90	0.97	0.93
m9	0.91	0.51	0.78	0.95	0.86	0.92	0.87	0.97	0.92
m10	0.90	0.54	0.81	0.94	0.85	0.87	0.86	0.97	0.92
m11	0.88	0.44	0.74	0.93	0.84	0.91	0.86	0.98	0.91
m12	0.96	0.72	0.91	0.98	0.96	0.96	0.97	0.98	0.96
m13	0.94	0.71	0.79	0.96	0.91	0.92	0.91	0.96	0.94
m14	0.91	0.52	0.74	0.96	0.89	0.91	0.90	0.97	0.93
m15	0.92	0.49	0.75	0.96	0.88	0.91	0.87	0.98	0.93
m16	0.92	0.51	0.75	0.95	0.87	0.91	0.86	0.96	0.93
m17	0.98	0.88	0.91	0.99	0.98	0.98	0.99	0.99	0.97
m18	0.92	0.67	0.87	0.97	0.91	0.93	0.92	0.98	0.92
m19	0.92	0.64	0.83	0.95	0.91	0.91	0.92	0.96	0.93
m20	0.90	0.50	0.81	0.94	0.88	0.91	0.86	0.97	0.91
m21	0.91	0.54	0.82	0.94	0.87	0.91	0.87	0.98	0.91
m22	0.98	0.83	0.92	0.99	0.99	0.98	0.99	0.99	0.98
m23	0.95	0.71	0.81	0.97	0.93	0.94	0.92	0.98	0.94
m24	0.94	0.61	0.80	0.97	0.92	0.94	0.90	0.98	0.94
m25	0.93	0.51	0.76	0.96	0.90	0.92	0.90	0.97	0.92
m26	0.91	0.52	0.73	0.95	0.90	0.91	0.89	0.98	0.93
m27	0.97	0.62	0.84	0.95	0.94	0.94	0.94	1.00	0.97
m28	0.97	0.72	0.59	0.98	0.92	0.92	0.97	0.91	0.86
m29	0.80	0.64	0.66	0.84	0.78	0.78	0.76	0.87	0.83
m30	0.94	0.63	0.61	0.97	0.94	0.95	0.91	0.95	0.91
**Avg $\pm$ SD**	**0.93 $\pm$ 0.04**	**0.62 $\pm$ 0.12**	**0.79 $\pm$ 0.08**	**0.96 $\pm$ 0.03**	**0.90 $\pm$ 0.05**	**0.92 $\pm$ 0.04**	**0.90 $\pm$ 0.05**	**0.97 $\pm$ 0.02**	**0.93 $\pm$ 0.03**
